# Multilevel information fusion for cryptographic substitution box construction based on inevitable random noise in medical imaging

**DOI:** 10.1038/s41598-021-93344-z

**Published:** 2021-07-12

**Authors:** Muhammad Fahad Khan, Khalid Saleem, Mohammed Ali Alshara, Shariq Bashir

**Affiliations:** 1 Department of Computer Sciences, Quaid-i-Azam University, Islamabad, Pakistan; 2 Department of Software Engineering, Foundation University Islamabad, Islamabad, Pakistan; 3 Department of Information Technology, College of Computer and Information Sciences, Imam Mohammad Ibn Saud Islamic University, Riyadh, Saudi Arabia; 4 DMPS Computer Science Section, The College of Arts and Sciences, University of Nizwa, Nizwa, Sultanate of Oman

**Keywords:** Mathematics and computing, Applied mathematics, Computer science

## Abstract

Block cipher has been a standout amongst the most reliable option by which data security is accomplished. Block cipher strength against various attacks relies on substitution boxes. In literature, extensively algebraic structures, and chaotic systems-based techniques are available to design the cryptographic substitution boxes. Although, algebraic and chaotic systems-based approaches have favorable characteristics for the design of substitution boxes, but on the other side researchers have also pointed weaknesses in these approaches. First-time multilevel information fusion is introduced to construct the substitution boxes, having four layers; Multi Sources, Multi Features, Nonlinear Multi Features Whitening and Substitution Boxes Construction. Our proposed design does not hold the weakness of algebraic structures and chaotic systems because our novel s-box construction relies on the strength of true random numbers. In our proposed method true random numbers are generated from the inevitable random noise of medical imaging. The proposed design passes all the substitution box security evaluation criteria including Nonlinearity, Bit Independence Criterion (BIC), Strict Avalanche Criterion (SAC), Differential Approximation Probability (DP), Linear Approximation Probability (LP), and statistical tests, including resistance to Differential Attack, Correlation Analysis, 2D, 3D histogram analysis. The outcomes of the evaluation criteria validate that the proposed substitution boxes are effective for block ciphers; furthermore, the proposed substitution boxes attain better cryptographic strength as compared to very recent state-of-the-art techniques.

## Introduction

Information security is the protection of information and information systems from unauthorized access and manipulation. Block cipher has been a standout amongst the most reliable options by which data security is accomplished^1,2^. Block ciphers are deterministic cryptographic algorithms that operate on a plaintext block of n bits, to produce a block of cipher text of m bits. Here n and m length are not necessarily to same. Block cipher contains repeating rounds of key mixing, permutation and substitution layers, which make it difficult for cryptanalysis or attacks. Data Encryption Standard (DES) and Advanced Encryption Standard (AES) are the most famous examples of block cipher. For block ciphers such as AES and DES, linear and differential cryptanalysis attacks are considered as powerful attacks. These cryptanalysis attacks are based on probabilistic characteristics of cipher parameters and output. Such an attack identifies the strength of the encryption algorithm by exponentially growing the number of rounds. For example, a differential attack examines how the differences in input reflect as differences in output. The ultimate goal of the attacker is to find the non-random behavior of the output which helps the attacker to attack the algorithm. For this purpose, attacker tries to put specific set of inputs to trace the differences in the output of every round. In linear cryptanalysis, the attacker tries to learn the probabilistic linear relations between the parity bits of plaintext, cipher text, and the key, by running the cipher several times^3,4^. Responsibility to make the correlation between cipher text and the key, as undetectable as possible, is on Substitution box (S-box). An S-box is an m x n mapping S: {0, 1}^m^ $\to${0, 1}^n^, transforming input vector x = [x_n−1_, x_n−2,_ x_n−3_ … x_0_] into output vector y = [y_n−1_, y_n−2,_ y_n−3_ … y_0_].

The noise of medical imaging which captured from multi sensors, multi-sources is an inevitable random phenomenon caused by the numerous intrinsic and inevitable switching factors including sensors temperature, spectral density, trap state, mobility fluctuation, charge trapping, free carriers, thermal motion of the ions, pulse sequence, field strength, charged particles scattering, optical density fluctuation and so on. Correlation and distribution of noise values captured from a single sensor, a single source of medical images is noticeably different from noise values that are captured from the multi-sensors, multi-sources. Noise captured from a single sensor and a single source has cohesiveness, more patterns, less uniform distribution compare to noise captured from the multi-sensors, multi-sources.

Remaining paper is organized as follows; "Contribution" section presents our core contribution; "Weaknesses in existing S-box designs" section describes weaknesses in existing s-box designs. "Proposed information fusion design" section explains the proposed multilevel information fusion for the construction of S-boxes. "Results and evaluation: S-box analysis" section presents the results and its evaluation; "Conclusion" section gives the concluding remarks.

### Contribution

The main contribution of this research is summarized in the following:

1. First-time multilevel information fusion is introduced to construct the cryptographic substitution boxes.
2. Extraction of true random numbers from the inevitable random noise of medical imaging, to synthesize the multi-level information fusion, for the construction of substitution boxes.
3. We introduce a novel nonlinear multi features whitening technique.
4. The proposed design passes all substitution box security evaluation criteria including Nonlinearity, Bit Independence Criterion (BIC), Strict Avalanche Criterion (SAC), Differential Approximation Probability(DP), Linear Approximation Probability(LP), and Statistical tests including resistance to Differential Attack, Correlation Analysis, 2D,3D histogram analysis.
5. The substitution-boxes constructed from our proposed design does not bear weaknesses of algebraic structures and chaotic systems.

### Weaknesses in existing S-box designs

In literature, extensively algebraic structures and chaotic systems-based methods are available for designing the S-boxes. Although algebraic and chaotic systems base approaches provide favourable characteristics for the S-box design, but researchers have also pointed weaknesses in these approaches. S-box designs of pure algebraic structures are jeopardized due to the intrinsic algebraic structure. Various algebraic attacks are available for algebraic construction of S-boxes including linear and differential attacks^3–17^, interpolation attacks^18–21^, Gröbner basis attack^22–28^, side-channel attacks^29–37^, SAT solver^38–45^, XSL attack^18,46–55^, XL attacks^56–60^.

Similarly, chaotic systems widely adopted in the designs of substitution boxes^61–74^. But due to the inherent algorithmic evolution of control parameters and periodic nature of chaotic maps, many weaknesses also exist in the literature, including discontinuity in chaotic sequences^1,67,75–78^, non-uniform distribution of chaotic sequences^1,75,76,79,80^, predictability^66,81–91^, finite precision effect^1,75,78,92,93^, dynamical degradation of chaotic systems^61,78,92–94^, small number of control parameters^79,82,83,95^, frail chaos^67,94^, short quantity of randomness^1,11,67,75–77,79,80,84,92,93,96–99^ Inherent intrinsic evolution of algebraic structures and its automorphism nature is an essential problem. Alternatively, researchers endorse the true random sequences for cryptography due to the fact that these sequences rely on the strength of naturally occurring processes to generate the true randomness^100–107^. True random sequences are irreversible, unpredictable, and unreproducible, even if their internal structure and response history are known to adversaries.

The objective of this research is extraction of true random numbers from the inevitable random noise of medical imaging, to synthesize the multi-level information fusion, for the construct of cryptographically strong substitution boxes.

## Proposed information fusion design

**Figure 1:**
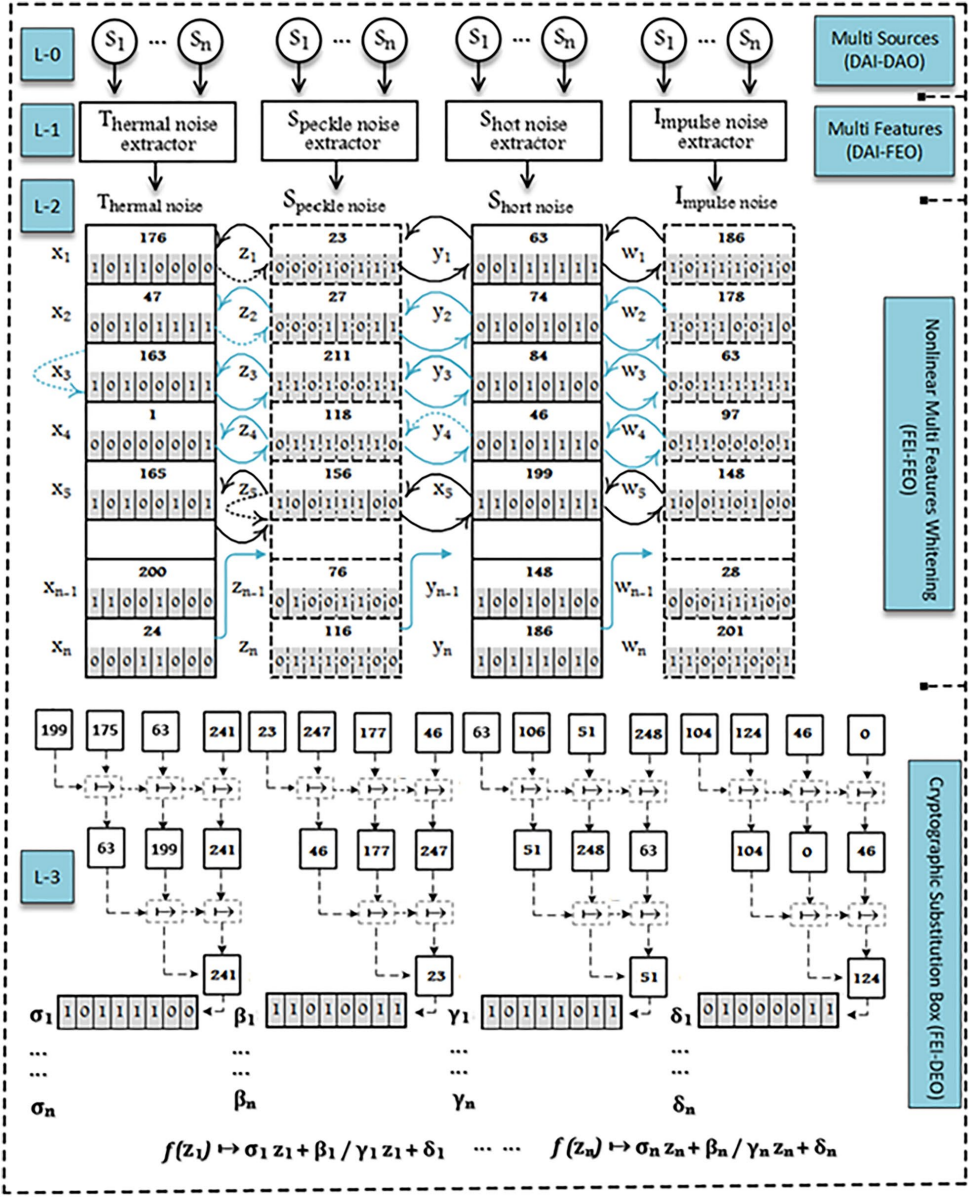
Multilevel information fusion for substitution boxes construction.

The proposed design of multilevel information fusion for substitution boxes construction have four layers Multi Sources, Multi Features, Nonlinear Multi Features Whitening and Substitution Boxes Construction. The whole design is thoroughly explained in the following layers and depicted in Fig. 1.

### Multi sources (L0: DAI-DAO)

This layer takes multiple medical imaging objects as input and gives raw objects to layer 1. One hundred medical imaging objects of random sources and random organs are taken from the UACI, Kaggle, SpineWeb, softnetaMedDream, OASIS, TCIA repositories.

### Multi features (L1: DAI-FEO)

Noise is an inevitable random phenomenon caused by the numerous intrinsic and inevitable switching factors including sensors temperature, spectral density, trap state, mobility fluctuation, charge trapping, free carriers, thermal motion of the ions, pulse sequence, field strength, charged particles scattering, optical density fluctuation and so on. In this layer primary four types of noise features are extracted from the raw objects of layer 1. First type is Thermal noise which is an inevitable energy equilibrium fluctuation phenomenon caused by the thermal agitation of electrons in resistances. Thermal noise always presents in medical imaging sensors and devices pre-eminently in multimodality imaging. The spectrum of thermal noise is flat over a wide range of frequencies. Features of the thermal noise extracted using^108,109^. Second is shot noise which is caused by the fact that current flowing across a junction isn’t smooth but is comprised of individual electrons arriving at random times. This non-uniform flow gives rise to broadband white noise that gets worse with increasing average. Features of the shot noise extracted using^106^. Third is speckle noise, which is a multiplicative noise that accompanies all coherent imaging modalities, in which images are produced by interfering echoes of a transmitted waveform and backscattered reflection. Forth is impulse noise, which is the most common noise that exists in medical modalities. Normally, impulse noise generated during the image acquisition, storage and transmission. In impulse noise some of the pixels are replaced by outliers while the rest remain unchanged. This layer gives features of these four noises to layer 2. Features of the speckle noise and impulse noise extracted using^88,110–112^ respectively.

### Nonlinear multi features whitening (L2: FEI-FEO)

Inherently various characteristics of the true randomness are existing in the noise features of medical imaging, which captured from multi sensors, multi-sources but these characteristics are unable to pass each NIST statistical randomness tests. In Table 1, we can see that various NIST randomness criteria failed, thus requiring a technique that is able to decrease the correlation, cohesiveness, and periodicity among multi-features, creating a highly random output that appears independent from multi-sources and uniformly distributed. To adhere to these requirements, we propose a technique called the “Nonlinear Multi Features Whitening”, which takes Multi features of the noise as input and returns uniformly distributed output that has no cohesiveness and calculable periodicity. The output of the proposed scheme has good statistical properties, including the elimination of statistical autocorrelation among multi-features. Thus, making the suggested multi-features fusion technique suitable for symmetric encryption.

**Table 1:** Noise features of medical imaging objects.

NIST tests	Noise features			
	Thermal	Speckle	Shot	Impulse
Frequency (Monobit) Test	Pass	Fail	Fail	Fail
Frequency Test within a Block	Fail	Fail	Fail	Fail
Runs Test	Fail	Fail	Fail	Pass
Longest Run of Ones in a Block	Fail	Pass	Fail	Fail
Binary Matrix Rank Test	Pass	Pass	Fail	Pass
Discrete Fourier Transform Test	Fail	Fail	Fail	Fail
Non-overlapping Template Matching Test	Fail	Pass	Fail	Fail
Overlapping Template Matching Test	Fail	Pass	Pass	Fail
Maurer’s Universal Statistical Test	Pass	Fail	Pass	Pass
Linear Complexity Test	Pass	Pass	Pass	Pass
Serial Test	Pass	Pass	Pass	Pass
Approximate Entropy Test	Fail	Fail	Fail	Fail
Cumulative Sums Test	Pass	Pass	Pass	Pass

The nonlinear multi features whitening design has two basic transformations: horizontal permutation and vertical permutation. This layer takes multi noise features including thermal, speckle, shot and impulse from the layer-1 and returns highly random output that appears independent from the input features. This layer also decreases the correlation, cohesiveness, and periodicity among the input features. The whole design of nonlinear multi features whitening thoroughly explained in the following steps and depicted in L2 of the Fig. 1.

#### Horizontal permutation

**Figure 2:**
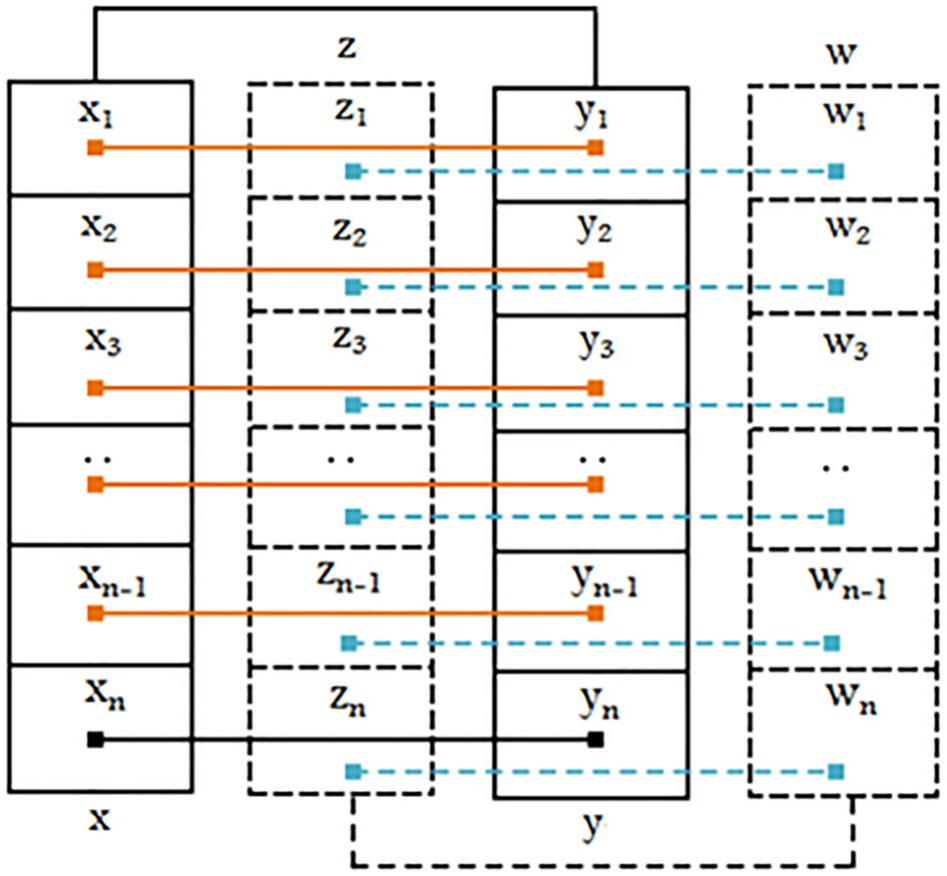
Pairs of vectors $x,y$ and vector $z,w$.

- *Step 1*: Convert Thermal noise $\left( {{\text{T}}_{{{\text{nf}}}} } \right)$, Speckle noise $\left( {{\text{Sp}}_{{{\text{nf~}}}} } \right)$, Shot $\left( {{\text{Sh}}_{{{\text{nf~}}}} } \right)$,Impulse noise $\left( {{\text{I}}_{{{\text{nf}}}} } \right)$ values into their respective binary representation. Where vector $x$ holds ${\text{T}}_{{{\text{nf}}}}$ binaries, vector $z$ holds ${\text{Sp}}_{{{\text{nf~}}}}$ binaries, vector $y$ holds ${\text{Sh}}_{{{\text{nf~}}}}$ binaries and $w$ holds ${\text{I}}_{{{\text{nf}}}}$ binaries.
- *Step 2*: Select the LSB of the $x_{i}$ and check if the selected bit is ‘0’ then execute step-3 to step-8 and if the selected bit is ‘1’ then execute step-9 to step-14. A visual representation of the vector $x,y$ pair and vector $z,w$ pair are depicted in Fig. 2.
- *Step 3*: If the frequency of 0's in leftmost 6 bits of $x_{i}$ are greater than frequency of 0's in leftmost 6 bits of ${\text{y}}_{{\text{i}}}$ then permute twice the $x_{i}$, $z_{i}$, $y_{i}$, $w_{i}$ from left to right respectively.
- *Step 4*: If the frequency of 0's in leftmost 6 bits of $x_{i}$ are less than frequency of 0's in leftmost 6 bits of $y_{i}$ then permute twice the $y_{i}$, $w_{i}$, $x_{i}$, $z_{i}$ from right to left respectively.
- *Step 5*: If the frequency of 0's in leftmost 6 bits of $x_{i}$ and leftmost 6 bits of $y_{i}$ are the same then execute step-6 or step-7, accordingly.
- *Step 6*: If the frequency of 0's in leftmost 6 bits of $z_{i}$ are greater than frequency of 0's in leftmost 6 bits of ${\text{w}}_{{\text{i}}}$ then permute twice the $z_{i}$, $y_{i}$, $w_{i}$, $x_{i}$ from left to right respectively.
- *Step 7*: If the frequency of 0's in leftmost 6 bits of $z_{i}$ are less than frequency of 0's in leftmost 6 bits of $w_{i}$ then permute twice the $w_{i}$, $y_{i}$, $z_{i}$, $x_{i}$ from right to left respectively.
- *Step 8*: If the frequency of 0's in leftmost 6 bits of $z_{i}$ and leftmost 6 bits of ${\text{w}}_{{\text{i}}}$ are same then discard the $x_{i}$, $z_{i}$, $y_{i}$, $w_{i}$.
- *Step 9*: If the frequency of 1's in leftmost 6 bits of $x_{i}$ are greater than frequency of 1's in leftmost 6 bits of ${\text{y}}_{{\text{i}}}$ then permute twice the $x_{i}$, $z_{i}$, $y_{i}$, $w_{i}$ from left to right, respectively.
- *Step 10*: If the frequency of 1's in leftmost 6 bits of $x_{i}$ are less than frequency of 1's in leftmost 6 bits of $y_{i}$ then permute twice the $y_{i}$, $w_{i}$, $x_{i}$, $z_{i}$ from right to left respectively.
- *Step 11*: If the frequency of 1's in leftmost 6 bits of $x_{i}$ and leftmost 6 bits of $y_{i}$ are the same then execute step-12 or step-13 accordingly.
- *Step 12*: If the frequency of 1's in leftmost 6 bits of $z_{i}$ are greater than frequency of 1's in leftmost 6 bits of ${\text{w}}_{{\text{i}}}$ then permute twice the $z_{i}$*,* $y_{i}$, $w_{i}$, $x_{i}$ from left to right respectively.
- *Step 13*: If the frequency of 1's in leftmost 6 bits of $z_{i}$ are less than frequency of 1's in leftmost 6 bits of $w_{i}$ then permute twice the $w_{i}$, $y_{i}$, $z_{i}$, $x_{i}$ from right to left respectively.
- *Step 14*: If the frequency of 1's in leftmost 6 bits of $z_{i}$ and leftmost 6 bits of ${\text{w}}_{{\text{i}}}$ are also same then discard the $x_{i}$, $z_{i}$, $y_{i}$, $w_{i}$.

#### Vertical permutation

**Figure 3:**
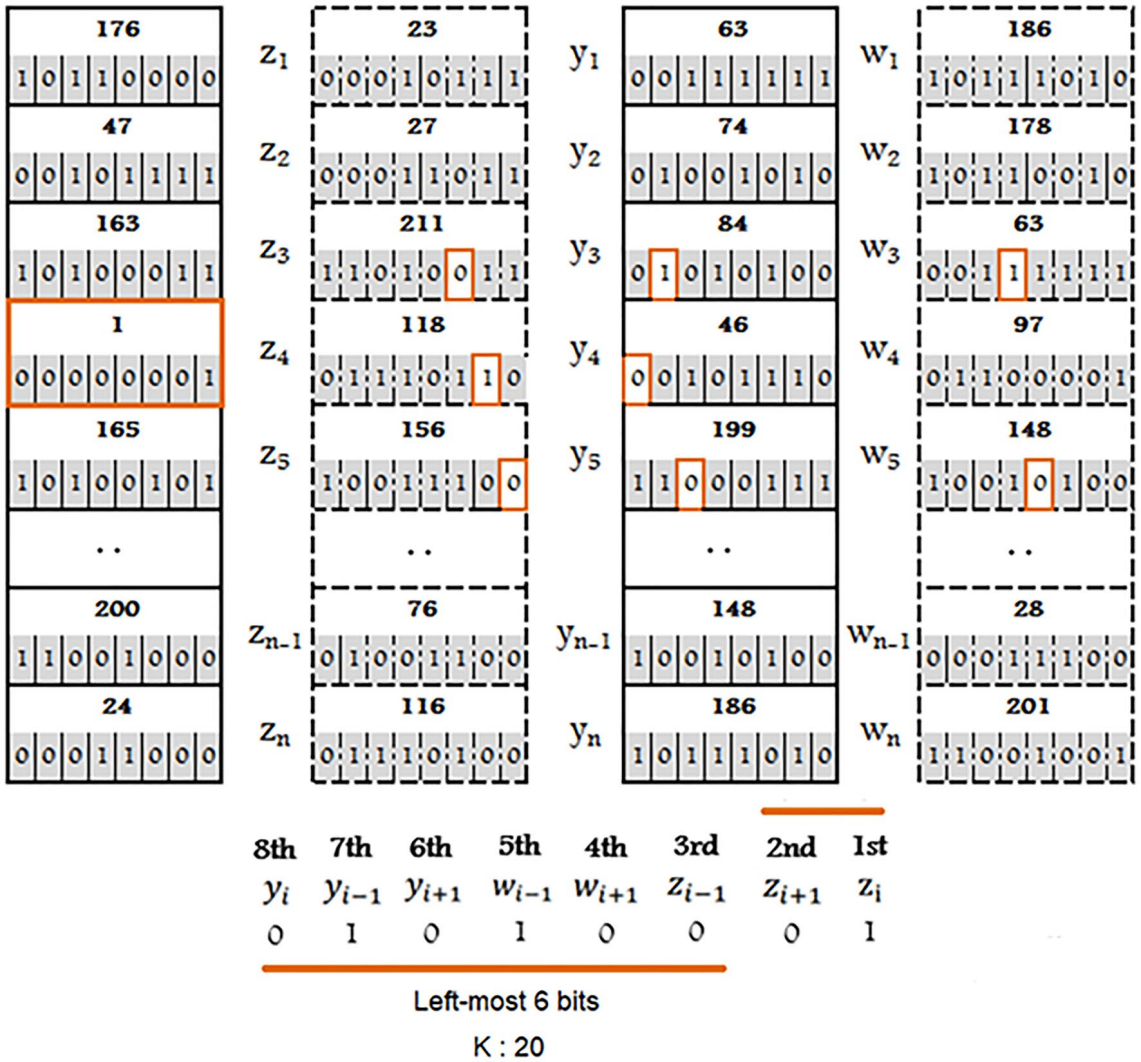
Vertical permutation.

- *Step 1*: Assign fused octet values according to the following:
  - 1st bit ← 2nd bit of ${\text{z}}_{{\text{i}}}$
  - 2nd bit ← 1st bit of $z_{{i + 1}}$
  - 3rd bit ← 3rd bit of $z_{{i - 1}}$
  - 4th bit ← 4th bit of $w_{{i + 1}}$
  - 5th bit ← 5th bit of $w_{{i - 1}}$
  - 6th bit ← 6th bit of $y_{{i + 1}}$
  - 7th bit ← 7th bit of $y_{{i - 1}}$
  - 8th bit ← 8th bit of $y_{i}$
    A visual representation of vertical permutation depicted in Fig. 3.
- *Step 2*: For variable ‘K’: Parse left-most 6 bits of the fused byte into corresponding decimal value.
- *Step 3*: If 1st bit of the fused octet is ‘0’ then K times cyclically permute the $column_{x}$ in the top to bottom manner.
- *Step 4*: If 1st bit of the fused octet is ‘1’ then K times cyclically permute the $column_{y}$ in the bottom to top manner.
- *Step 5*: If 2nd bit of the fused octet is ‘0’ then K times cyclically permute the $column_{z}$ in the top to bottom manner.
- *Step 6*: If 2nd bit of the fused octet is ‘1’ then K times cyclically permute the $column_{w}$ in the bottom to top manner.

### Substitution boxes construction (L3: FEI-DEO)

**Figure 4:**
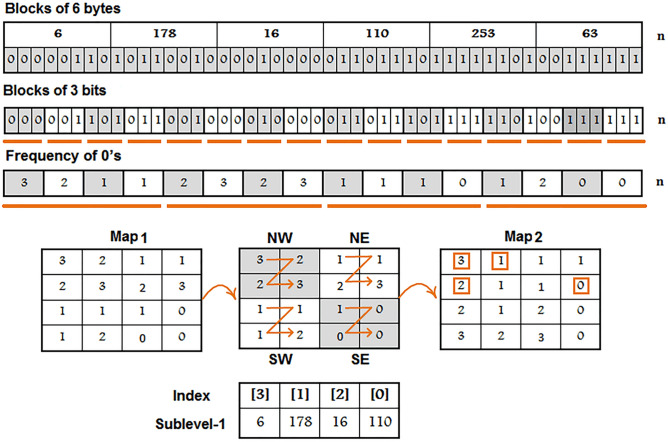
Visual representation of steps 2 to 11.

**Figure 5:**
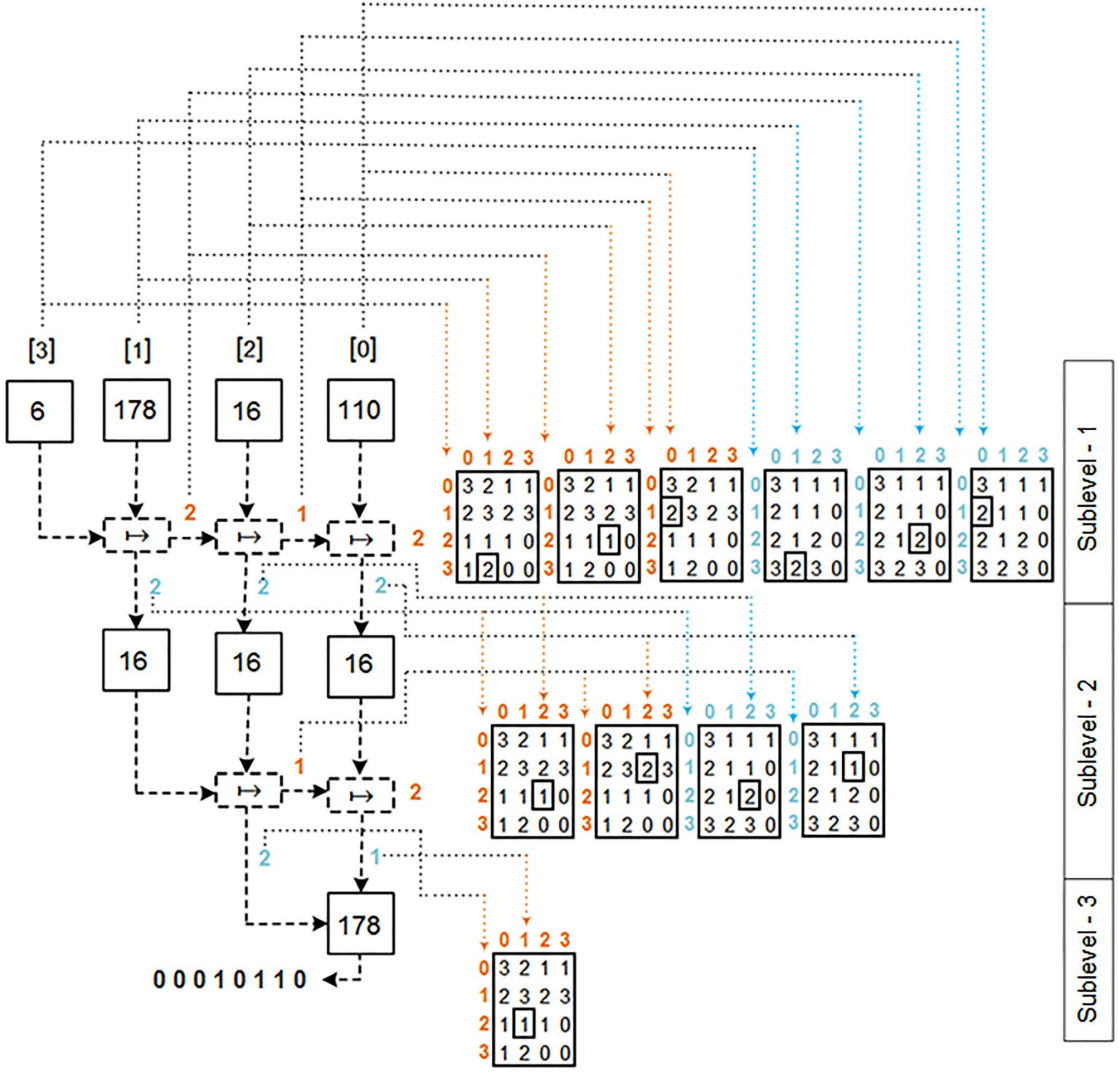
Visual representation of steps 15 to 19.

This layer takes highly correlated true random numbers from the L2 and generates substitution boxes. The complete design of this layer is thoroughly described in the following steps:

- *Step 1*: Traverse each value of $column_{x}$, ${\text{~}}column_{y}$, ${\text{~}}column_{z}$, and ${\text{~}}column_{w}$, row by row and then concatenate the subsequent rows in a one dimensional vector. A visual representation of steps 2 to 11 is depicted in Fig. 4.
- *Step 2*: Split one-dimensional vector into blocks of 6 bytes. If the length of last block is less than 6 then exclude last block.
- *Step 3*: Convert each block of step-2 into binary representation and then split into blocks of 3 bits.
- *Step 4*: Get frequency of 0’s in every block which are obtained from step-3.
- *Step 5*: Consider the block-0 to block-3 values of step-4 as the 1st row of matrix Map^1^.
- *Step 6*: Consider the block-4 to block-7 values of step-4 as the 2nd row of matrix Map^1^.
- *Step 7*: Consider the block-8 to block-11 values of step-4 as the 3rd row of matrix Map^1^.
- *Step 8*: Consider the block-12 to block-15 values of step-4 as the 4th row of matrix Map^1^.
- *Step 9*: To create the matrix Map^2^ : Traverse Map^1^ in the Z-order format. Consider quadrant NW as a 1st column, quadrant NE as a 3rd column, quadrant SW as 2nd column and quadrant SE as a 4th column of Map^2^.
- *Step 10*: Traverse first 4 unique values of Map^2^ from row-major order.
- *Step 11*: Sublevel-1 indexes assignment: Consider the values of step-10 as indexes of the first-4 unique elements which are obtained from step-2. A visual representation of the steps 12 to 14 is shown in the sublevel-1 of Fig. 5.
- *Step 12*: To Get 1st $right_{{index}}$: Use each index of the left most block to select a row of Map^1^ and the second left-most index of a block to select a column of Map^1^.
- *Step 13*: To Get 2nd $right_{{index}} {\text{~}}$: For first parameter, take index from step-12 and for second parameter, take the index of third left-most element which gained from step-11. Use first and second parameters to select a row and column of Map^1^.
- *Step 14*: To Get 3rd $right_{{index}}$: For first parameter, take index from step-13 and for second parameter, take the index of fourth left-most element which gained from step-11. Use first and second parameters to select a row and column of Map^1^.
- *Step 15*: Get 1st permutated element of sublevel-2: To obtain index, execute the step-12 over Map^2^, except Map^1^, and get permutated element through the retrieved index. A visual representation of the steps 15 to 19 is shown in the sublevel-2 of Fig. 5.
- *Step 16*: Get 2nd permutated element of sublevel-2: To obtain index, execute the step-13 over Map^2^, except Map^1^, and get permutated element through the retrieved index.
- *Step 17*: Get 3rd permutated element of sublevel-2: To obtain index, execute the step-14 over Map^2^, except Map^1^, and get permutated element through the retrieved index.
- *Step 18*: To Get 4th $right_{{index}} {\text{~}}$: For first parameter, take the index of output of the step-15 and for second parameter, take the index of output of the step-16. Use first and second parameter to select a row and column of Map^1^.
- *Step 19*: To Get 5th $right_{{index}} {\text{~}}$: For first parameter, take the index of output of the step-18. For second parameter, take the index of output of the step-17. Use first and second parameters to select a row and column of Map^1^.
- *Step 20*: Get 1st permutated element of sublevel-3: To obtain index, execute the step-14 over Map^2^, except Map^1^, by passing resultant values of step-18 and step-19 as parameters. Fetch permutated element through the retrieved index.
- *Step 21*: To Get 6th $right_{{index}} {\text{~}}$: Take index of the 1st permutated element of sublevel-3
- *Step 22*: To acquire arbitrary bits of permutated elements: Obtain indexes from step-12 to step-14, step-18 to step-19, and step-21. Acquire the respective bits of permutated elements from these indexes.
- *Step 23*: Get a most significant bit and least significant bit from the permutated element of step-15.
- *Step 24*: Subsequently, generate σ_*i*_, β_*i*_, γ_*i*_, δ_*i*_: Concatenate the bits of step-22 and step-23 thus parse these bits into their particular decimal value.
- *Step 25*: Put values of σ_*i*_, β_*i*_, γ_*i*_, δ_*i*_ in linear fraction transform f ($z_{i}$) $\mapsto$ (σ_*i*_ $z_{i}$ + β_*i*_)/(γ_*i*_ $z_{i}$ + δ_1_) where γ_*i*_ $z_{i}$ =  − δ_1_ and σ_*i*_δ_1_ − β_*i*_γ_*i*_, ≠ 0. Remove repeating elements from the f ($z_{i}$) block of 256 size and transformed elements in a s-box of size 8 × 8.

## Results and evaluation: S-box analysis

This section covers the results and evaluation of the results. The resultant S-boxes evaluated through the standard S-box evaluation criteria^65,69,74,113–124^ including Strict Avalanche Criterion (SAC), Bit Independence Criterion (BIC), Linear Approximation Probability(LP), Differential Approximation Probability(DP), Nonlinearity, and through the statistical tests including resistance to Differential Attack, Correlation Analysis, 2D, 3D histogram analysis. From the proposed design the total number of 4562 S-boxes constructed, where the nonlinearity of 821 S-boxes are less-than 109, the nonlinearity of 3728 S-boxes are equal to 110 and the nonlinearity of 13 S-boxes is equal to 112. Two S-boxes picked randomly from the resultant S-boxes as samples and shown in the Table 2a, b. The nonlinearity of S-boxes which are shown in Table 2a, b are 110 and 112 respectively.

**Table 2:** (a) Substitution box 1. (b) Substitution box 2.

a															
230	219	63	165	50	128	59	206	187	117	181	92	156	248	159	116
44	102	202	21	254	170	13	176	246	33	160	91	56	11	173	153
141	185	15	93	178	127	207	140	38	245	12	157	214	168	225	183
163	139	68	213	98	134	154	58	10	162	175	119	136	107	66	223
221	198	238	1	199	155	169	97	212	28	234	110	188	233	255	69
200	151	148	251	112	104	236	125	32	80	239	218	53	65	243	43
42	149	121	61	18	228	90	204	220	76	250	130	195	194	26	232
113	115	30	242	101	215	25	158	126	189	54	197	55	120	186	111
129	123	209	17	78	48	94	108	49	34	166	150	86	0	152	45
4	8	167	210	171	138	161	192	249	81	89	177	2	147	231	96
142	60	20	100	51	71	64	145	205	103	84	146	211	7	241	72
29	99	3	16	217	23	82	244	133	226	83	144	224	9	27	240
216	193	105	172	6	135	62	203	57	88	184	252	87	227	196	190
208	24	41	47	36	247	109	79	201	19	191	131	237	235	67	70
46	253	22	31	35	73	174	164	180	75	14	5	124	106	114	39
118	143	122	229	222	37	52	179	74	85	137	132	182	40	77	95
**b**															
2	101	41	39	222	60	44	105	251	82	98	135	6	61	229	111
159	32	169	4	94	119	107	25	87	130	194	245	136	118	192	129
16	171	209	148	146	5	140	125	228	252	9	46	65	181	96	48
132	199	160	58	38	218	233	232	200	255	214	184	250	128	33	153
23	231	3	120	68	249	75	12	113	17	72	166	15	49	97	127
238	28	67	237	247	172	59	227	215	37	52	91	161	196	193	241
30	164	1	206	55	157	242	212	99	78	197	144	20	150	79	189
19	243	126	239	205	11	63	54	253	145	85	202	248	110	86	175
225	236	174	114	88	102	36	92	155	186	24	226	211	154	43	220
177	210	35	187	170	74	179	124	201	83	77	71	191	13	176	188
235	178	163	108	133	141	183	106	31	216	180	195	29	57	90	8
76	254	89	109	42	203	158	45	73	93	123	167	240	168	104	221
0	152	230	198	95	69	27	18	131	70	7	50	134	80	143	112
51	173	234	137	116	162	219	21	207	185	10	66	103	139	149	208
217	204	22	84	62	47	190	151	223	246	53	244	147	182	122	56
138	14	142	64	100	117	224	156	213	121	81	34	26	115	165	40

### Nonlinearity

The nonlinearity is the ability of substitution box that provides immunity from the linear cryptanalysis and it is exhibited by the nonlinearity score^1,128,129,142,145,154,155,158–175^. The nonlinearity of a substitution box h(x) described as the Walsh spectrum (WS):

1 $$ N_{h}  = 2^{{m - 1}} \left( {1 - 2^{{ - m}} ~~max~\left| {S_{{\left( g \right)}} \left( w \right)} \right|} \right) $$

The WS, h(x) defined as:

2 $$ S_{{\left\langle h \right\rangle }} \left( x \right) = \mathop \sum \limits_{{{\text{x~}} \in {\text{~GF}}\left( {2^{n} } \right)}}^{n} \left( { - 1)^{{h\left( x \right) \oplus x \cdot X}} } \right) $$

where ${\text{x}} \in {\text{GF}}\left( {2^{n} } \right)$ and $x \cdot X$ is the dot product of $x$ and $~X$, which is defined as:

3 $$ x \cdot X = x_{1}  \oplus X_{1}  + x_{2}  \oplus X_{2}  +  \cdots  + x_{{\text{n}}}  \oplus X_{{\text{n}}} $$

The nonlinearity score of substitution box-1 and substitution box-2 is 110 and 112 respectively. We observed that the Nonlinearity score of both these S-boxes are better than to the thirty state-of-the-art (2018 to 2020 year) S-boxes, mentioned in Table 3.

**Table 3:** Nonlinearity of the state-of-the-art S-boxes.

Substitution box	Max. nonlinearity	Substitution box	Max. nonlinearity
Özkaynak^130^, 2020	104	Azam^143^, 2019	108
El-Latif^131^, 2020	104	Özkaynak^120^, 2019	108
Zahir^132^, 2020	104	Hussam^144^, 2019	108
El-Latif^133^, 2020	106	Amjad^157^, 2019	108
Artuğer^134^, 2020	107	Özkaynak^46^, 2018	106
D. Lambić^135^, 2020	108	Tian^102^, 2018	106
H. Liu^136^, 2020	108	Silva^74^, 2018	106
Zhang^137^, 2020	108	Attaullah^146^, 2018	107
Cassal^138^, 2020	108	Alzaidi^147^, 2018	107
Bin^139^, 2020	104	Alzaidi^148^, 2018	107
KM^124^, 2019	106	Solami^149^, 2018	108
Tanyildizi^140^, 2019	106	Hayat^150^, 2018	108
Açikkapi^141^, 2019	106	Wang^151^, 2018	108
Özkaynak^72^, 2019	107	Liu^152^, 2018	108
Zahid^156^, 2019	108	Zhimao^153^, 2018	108

### Bit independence criterion (BIC)

One of the desirable characteristic of substitution box is bit independent criterion. BIC property is defined as all the avalanche variables should be pair-wise independent for a given set of avalanche vectors, created by complementing of only one plaintext bit. Bit independent criterion is examined by altering every input bit from the plaintext^1^. Suppose Boolean functions are S1, S2, . . . Sn and if two Boolean functions output bits, Sj and Sk satisfy BIC, the nonlinearity and SAC must be met by Sj ⊕ Sk (j ≠ k, 1 ≤ j, k ≤ n). The average of BIC-SAC matrix of the S-box1 is 0.499 and the S-box2 is also 4.99, which is very close to 0.5. Result shows that our proposed S-boxes are competent enough to fulfil the bit independent criteria. BIC results of the S-box1 and S-box2 are presented in Table 4a, b, respectively.

**Table 4:** (a) Bit independence criterion of the S-box1. (b) Bit independence criterion of the S-box2.

a							
–	0.507812	0.460938	0.503906	0.503906	0.513672	0.500000	0.482422
0.507812	–	0.476562	0.480469	0.482422	0.503906	0.511719	0.513672
0.460938	0.476562	–	0.474609	0.462891	0.513672	0.525391	0.535156
0.503906	0.480469	0.474609	–	0.509766	0.525391	0.507812	0.496094
0.503906	0.482422	0.462891	0.509766	–	0.503906	0.498047	0.517578
0.513672	0.503906	0.513672	0.525391	0.503906	–	0.490234	0.462891
0.500000	0.511719	0.525391	0.507812	0.498047	0.490234	–	0.482422
0.482422	0.513672	0.535156	0.496094	0.517578	0.462891	0.482422	–
**b**							
–	0.480469	0.492188	0.496094	0.492188	0.503906	0.519531	0.515625
0.480469	–	0.490234	0.482422	0.486328	0.484375	0.490234	0.505859
0.492188	0.490234	–	0.484375	0.519531	0.527344	0.515625	0.517578
0.496094	0.482422	0.484375	–	0.500000	0.488281	0.480469	0.521484
0.492188	0.486328	0.519531	0.500000	–	0.513672	0.478516	0.507812
0.503906	0.484375	0.527344	0.488281	0.513672	–	0.468750	0.470703
0.519531	0.490234	0.515625	0.480469	0.478516	0.468750	–	0.533203
0.515625	0.505859	0.517578	0.521484	0.507812	0.470703	0.533203	–

### Strict avalanche criterion (SAC)

The confusion strength of the substitution box is examined through the strict avalanche criteria. SAC determines how many output bits changed when a single change is made in input^1^. It is defined as: $f:{\text{~}}F_{2}^{n}  \to F_{2}$ satisfies if $f\left( x \right) \oplus f\left( {x \oplus \alpha } \right)$ is balanced for α = 1. Variance, Maximum, Minimum and Average SAC results of the S-box1 are 0.041869, 0.593750, 0.390625 and 0.503174, respectively. Variance, Maximum, Minimum and Average SAC results of the S-box2 are 0.032103, 0.578125, 0.437500 and 0.500488, respectively. The average SAC value of S-box1 and Sbox-2 dependency matrix are 0.503174 and 0.500488 which are exactly same to the ideal SAC. So, our proposed S-boxes satisfy the avalanche criteria. S-box1 and S-box2 results of the SAC are presented in Table 5a, b, respectively.

**Table 5:** (a) Strict Avalanche Criterion of the S-box1. (b) Strict Avalanche Criterion of the S-box2.

a							
0.500000	0.468750	0.531250	0.468750	0.468750	0.500000	0.500000	0.546875
0.484375	0.500000	0.515625	0.562500	0.531250	0.593750	0.562500	0.546875
0.531250	0.546875	0.500000	0.515625	0.500000	0.531250	0.515625	0.468750
0.437500	0.531250	0.515625	0.515625	0.453125	0.421875	0.500000	0.578125
0.546875	0.484375	0.500000	0.453125	0.531250	0.531250	0.578125	0.437500
0.468750	0.500000	0.531250	0.500000	0.390625	0.546875	0.468750	0.468750
0.453125	0.546875	0.500000	0.562500	0.453125	0.453125	0.546875	0.500000
0.515625	0.500000	0.546875	0.468750	0.500000	0.453125	0.437500	0.484375
**b**							
0.515625	0.515625	0.484375	0.515625	0.500000	0.453125	0.468750	0.484375
0.453125	0.453125	0.468750	0.515625	0.578125	0.562500	0.515625	0.531250
0.531250	0.531250	0.500000	0.453125	0.531250	0.468750	0.484375	0.531250
0.531250	0.453125	0.515625	0.484375	0.484375	0.500000	0.531250	0.515625
0.546875	0.500000	0.484375	0.468750	0.484375	0.562500	0.515625	0.500000
0.515625	0.531250	0.468750	0.546875	0.453125	0.500000	0.546875	0.500000
0.453125	0.515625	0.453125	0.468750	0.515625	0.515625	0.531250	0.484375
0.515625	0.437500	0.484375	0.515625	0.453125	0.500000	0.515625	0.484375

### Linear approximation probability (LP)

The imbalance of an event among input and output bit is identified by LP^79,80^. LP is the biggest disparity of an event, in which parity of the output bits is equal to the parity of the input bits. Here parity of the input bits is selected by the mask Ωx and parity of the output bits is selected by the mask Ωy.^56,74^. 2^n^ is the number of elements and X is the set of all feasible inputs. Maximum LP of our S-box1 is 0.140625 and S-box2 is 0.1171875, which satisfy the LP criteria.

4 $$ LP_{f}  = max_{{\Omega x,\Omega y \ne 0}} \left| {\frac{{\left\{ {\left. {x \in X} \right|x \cdot \Omega x = {\text{S}}\left( {\text{x}} \right) \cdot \Omega {\text{y}}} \right\}}}{{2^{n} }} - \frac{1}{2}} \right| $$

### Differential approximation probability (DP)

Differential uniformity of substitution box is measured through the differential approximation probability. For any change in the input either sequence or value, there has to be a change in the output. In DP, the input differential Δ $x_{i}$ should uniquely map to an output differential Δ $y_{i}$, therefore ensuring a uniform mapping probability for each $i$. In Table 6a, b we can see that the results of our S-box1 and S-box2 fully fills the DP criteria, represented as follows^1^:

5 $$ DP~\left( {\Delta x \to \Delta y} \right) = \left[ {\frac{{\# \{ \left. {x \in X} \right|({\text{S~}}\left( {\text{x}} \right) \oplus S\left( {x \oplus \Delta x} \right) = \Delta y\} }}{{2^{n} }}} \right] $$

**Table 6:** (a) DP of the S-box1. (b) DP of the proposed S-box2.

a															
0.00000	0.03125	0.02343	0.02343	0.03125	0.03125	0.02343	0.02343	0.02343	0.02343	0.03125	0.02343	0.02343	0.03125	0.02343	0.03125
0.02343	0.02343	0.03125	0.02343	0.02343	0.02343	0.02343	0.02343	0.02343	0.02343	0.03120	0.01562	0.02343	0.03906	0.03125	0.02343
0.03125	0.02343	0.03125	0.02343	0.02343	0.03125	0.03125	0.03125	0.02343	0.03125	0.02343	0.02343	0.03125	0.02343	0.03125	0.03125
0.02343	0.03906	0.02343	0.03125	0.02343	0.02343	0.01562	0.02343	0.02343	0.03125	0.02343	0.02343	0.03906	0.02343	0.03125	0.03125
0.02343	0.03125	0.03125	0.02343	0.02343	0.02343	0.03125	0.02343	0.03125	0.03125	0.03125	0.03125	0.02343	0.02343	0.02343	0.01562
0.02343	0.02343	0.02343	0.03125	0.03125	0.03125	0.02343	0.03125	0.02343	0.03125	0.03125	0.02343	0.02343	0.02343	0.02343	0.03125
0.03125	0.02343	0.02343	0.03125	0.03125	0.01562	0.03125	0.02343	0.02343	0.03906	0.02343	0.02343	0.03125	0.02343	0.03125	0.03125
0.03906	0.02343	0.02343	0.03125	0.03125	0.03125	0.03125	0.02343	0.03906	0.02343	0.03125	0.02343	0.02343	0.02343	0.02343	0.02343
0.03906	0.03125	0.02343	0.02343	0.01562	0.02343	0.03125	0.02343	0.03125	0.03125	0.03125	0.02343	0.02343	0.02343	0.03125	0.02343
0.02343	0.02343	0.02343	0.03125	0.03125	0.02343	0.02343	0.03125	0.03125	0.02343	0.03125	0.02343	0.03125	0.02343	0.03125	0.03125
0.03125	0.03125	0.02343	0.02343	0.02343	0.02343	0.03125	0.02343	0.03906	0.03125	0.04687	0.02343	0.02343	0.03125	0.02343	0.02343
0.02343	0.03125	0.03125	0.02343	0.03125	0.02343	0.02343	0.03125	0.02343	0.02343	0.03125	0.02343	0.03125	0.03125	0.03125	0.02343
0.03125	0.03125	0.03125	0.02343	0.02343	0.02343	0.03125	0.02343	0.02343	0.02343	0.02343	0.03125	0.02343	0.03125	0.02343	0.03125
0.02343	0.02343	0.02343	0.02343	0.03125	0.02343	0.03125	0.02343	0.02343	0.02343	0.01562	0.03125	0.02343	0.01562	0.03125	0.02343
0.02343	0.03125	0.02343	0.02343	0.02343	0.03125	0.03125	0.03125	0.03125	0.05468	0.02343	0.02343	0.03125	0.02343	0.02343	0.02343
0.02343	0.02343	0.02343	0.02343	0.03125	0.02343	0.02343	0.03125	0.02343	0.02343	0.02343	0.02343	0.03125	0.03906	0.02343	0.02343
**b**															
0.00000	0.02343	0.03906	0.02343	0.02343	0.02343	0.02343	0.03125	0.03125	0.02343	0.03125	0.02343	0.02343	0.02343	0.03125	0.02343
0.03125	0.02343	0.03125	0.03125	0.03125	0.02343	0.02343	0.02343	0.02343	0.02343	0.02343	0.03125	0.02343	0.02343	0.02343	0.02343
0.02343	0.03125	0.02343	0.02343	0.02343	0.03125	0.03125	0.02343	0.02343	0.02343	0.02343	0.03125	0.03125	0.02343	0.02343	0.03125
0.02343	0.03125	0.03125	0.02343	0.02343	0.03906	0.02343	0.03125	0.03125	0.02343	0.02343	0.02343	0.03125	0.03125	0.02343	0.02343
0.02343	0.03125	0.02343	0.02343	0.02343	0.02343	0.03125	0.02343	0.02343	0.02343	0.01562	0.03125	0.02343	0.02343	0.02343	0.03125
0.03125	0.02343	0.02343	0.02343	0.02343	0.03906	0.02343	0.02343	0.02343	0.03125	0.02343	0.03906	0.02343	0.03125	0.02343	0.02343
0.03125	0.03125	0.02343	0.02343	0.02343	0.03125	0.02343	0.02343	0.03906	0.02343	0.03125	0.02343	0.02343	0.02343	0.03125	0.02343
0.02343	0.02343	0.03125	0.02343	0.03125	0.02343	0.02343	0.03125	0.02343	0.02343	0.02343	0.02343	0.02343	0.02343	0.03906	0.02343
0.03125	0.02343	0.03906	0.03906	0.03125	0.02343	0.02343	0.02343	0.02343	0.02343	0.02343	0.02343	0.02343	0.03125	0.03125	0.02343
0.02343	0.02343	0.02343	0.03125	0.02343	0.02343	0.02343	0.02343	0.02343	0.02343	0.03125	0.02343	0.02343	0.03125	0.03906	0.02343
0.03125	0.02343	0.02343	0.02343	0.03125	0.02343	0.02343	0.02343	0.02343	0.02343	0.03906	0.02343	0.02343	0.02343	0.02343	0.02343
0.02343	0.02343	0.02343	0.02343	0.03125	0.02343	0.02343	0.03125	0.02343	0.02343	0.02343	0.02343	0.02343	0.03125	0.03125	0.03125
0.02343	0.03125	0.02343	0.02343	0.03125	0.02343	0.02343	0.02343	0.03125	0.02343	0.02343	0.03125	0.03125	0.02343	0.02343	0.02343
0.03125	0.02343	0.02343	0.02343	0.02343	0.03906	0.02343	0.02343	0.02343	0.03125	0.02343	0.03125	0.03125	0.02343	0.03125	0.03125
0.03125	0.03125	0.03125	0.02343	0.02343	0.02343	0.02343	0.02343	0.02343	0.02343	0.02348	0.02343	0.02343	0.02343	0.02343	0.02343
0.03125	0.03125	0.03125	0.03125	0.02343	0.02343	0.02343	0.02343	0.02343	0.03125	0.02343	0.02343	0.03125	0.03906	0.03125	0.03125

### Evaluation: statistical analysis

#### Resistance to differential attack

Number of changing pixel rate (NPCR) and Unified Averaged Changed Intensity (UACI) are the performance indicators that have the ability to assess the resilience of cipher against differential attacks^9,74^. Mathematically they are defined in Eqs. (6) and (7).

6 $$ NPCR~ = \frac{{\mathop \sum \nolimits_{{i,j}} ~D\left( {i,j} \right)}}{{N \times M}} \times 100\% $$

7 $$ UACI~ = \frac{1}{{N \times M}} \times \left[ {\mathop \sum \limits_{{i,j}}  \times \frac{{\left| {C_{1} \left( {i,j} \right) - C_{2} \left( {i,j} \right)} \right|}}{{255}}} \right] \times 100\% $$

In Eq. (8) $C_{1} \left( {i,j} \right)$ and $C_{2} \left( {i,j} \right)$ are two encrypted images obtained from plaintext images that are slightly different. where ${\text{N}}$ is the width, ${\text{M}}$ is height of the encrypted image and $D\left( {i,j} \right)$ is the difference function between encrypted images. Difference is given as:

8 $$ f\left( x \right) = \left\{ {\begin{array}{*{20}l}    {0,} \hfill & {{\text{if}}\;{\text{C}}_{1} \left( {{\text{i}},{\text{j}}} \right) = {\text{C}}_{2} \left( {{\text{i}},{\text{j}}} \right),} \hfill  \\    {1,} \hfill & {{\text{if}}\;{\text{C}}_{1} \left( {{\text{i}},{\text{j}}} \right) \ne {\text{C}}_{2} \left( {{\text{i}},{\text{j}}} \right),} \hfill  \\   \end{array} } \right. $$

To evaluate the impact of pixels change, in the plain images over the encrypted images various test images are evaluated through the NPCR and UACI measures. Experimental results of the NPCR and UACI for each channel (R, G, B) of baboon, cameraman,parrot and fruits are presented in Table 7. Constantly NPCR values of our results are close to 99.9 which is the perfect value. Similarly, all the values of UACI are greater than 33.5 which is also the perfect value.

**Table 7:** NPCR and UACI.

Images	Location	NPCR	UACI
Baboon	Last	99.82	33.47
	Mid	99.83	33.43
	First	99.87	33.45
Cameraman	Last	99.85	33.49
	Mid	99.78	33.52
	First	99.84	33.55
Parrot	Last	99.85	33.42
	Mid	99.83	33.46
	First	99.81	33.45
Fruits	Last	99.91	33.58
	Mid	99.89	33.59
	First	99.82	33.51

### Histogram analysis

Here histogram analysis is used to examine the resistance of encryption algorithm against the statistical attacks. It shows, how plain image pixels scatter after the encryption process and it indicates the frequency distribution of encrypted pixels. Ideal encryption technique reconstructs the plain image into encrypted image that bears the random pixel values and sequences. We examined the 2D and 3D histograms of various color images.

**Figure 6:**
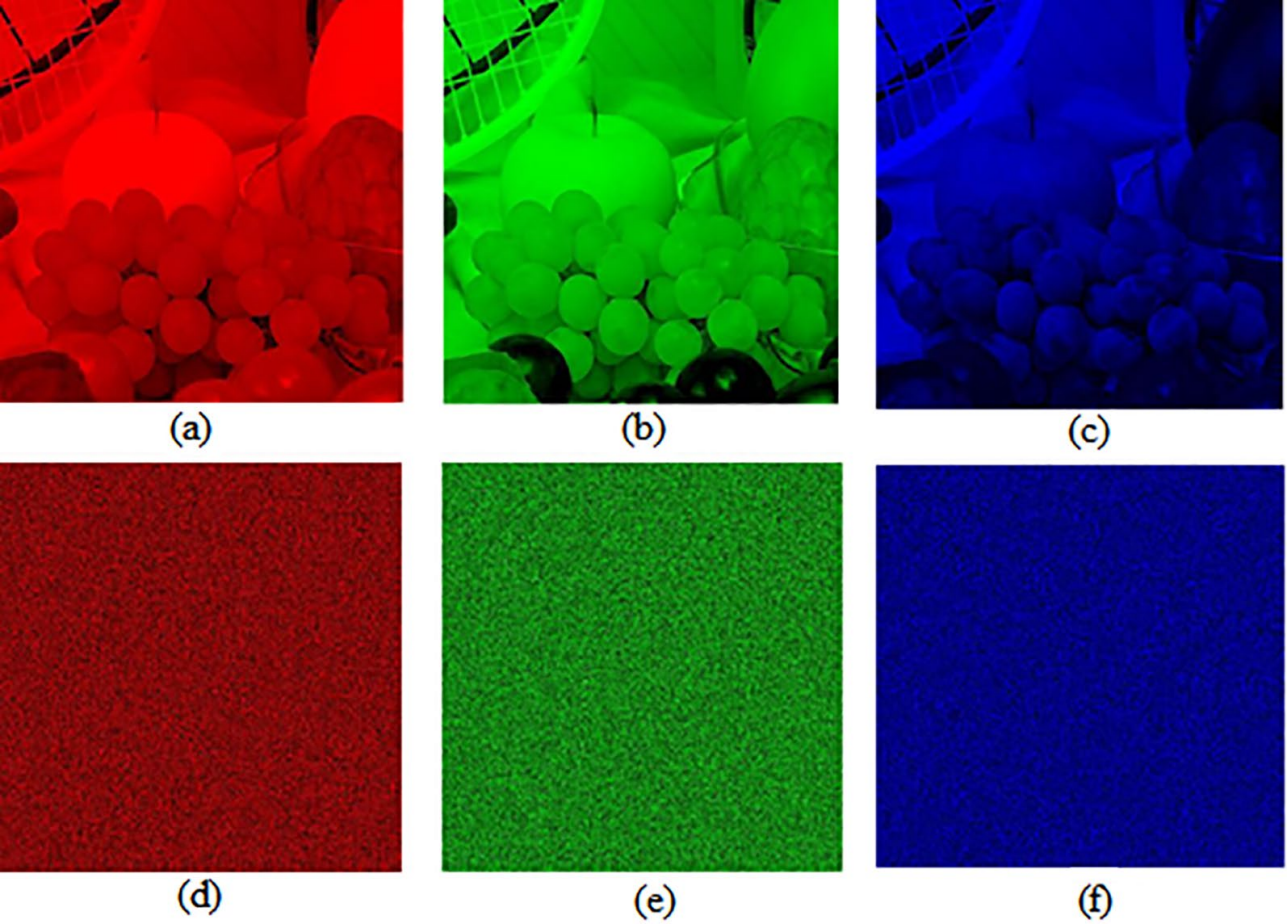
Plain and encrypted images of fruit. (**a**) R channel; (**b**) G channel; (**c**) B channel; (**d**) Encrypted R channel; (**e**) Encrypted G channel; (**f**) Encrypted B channel.

**Figure 7:**
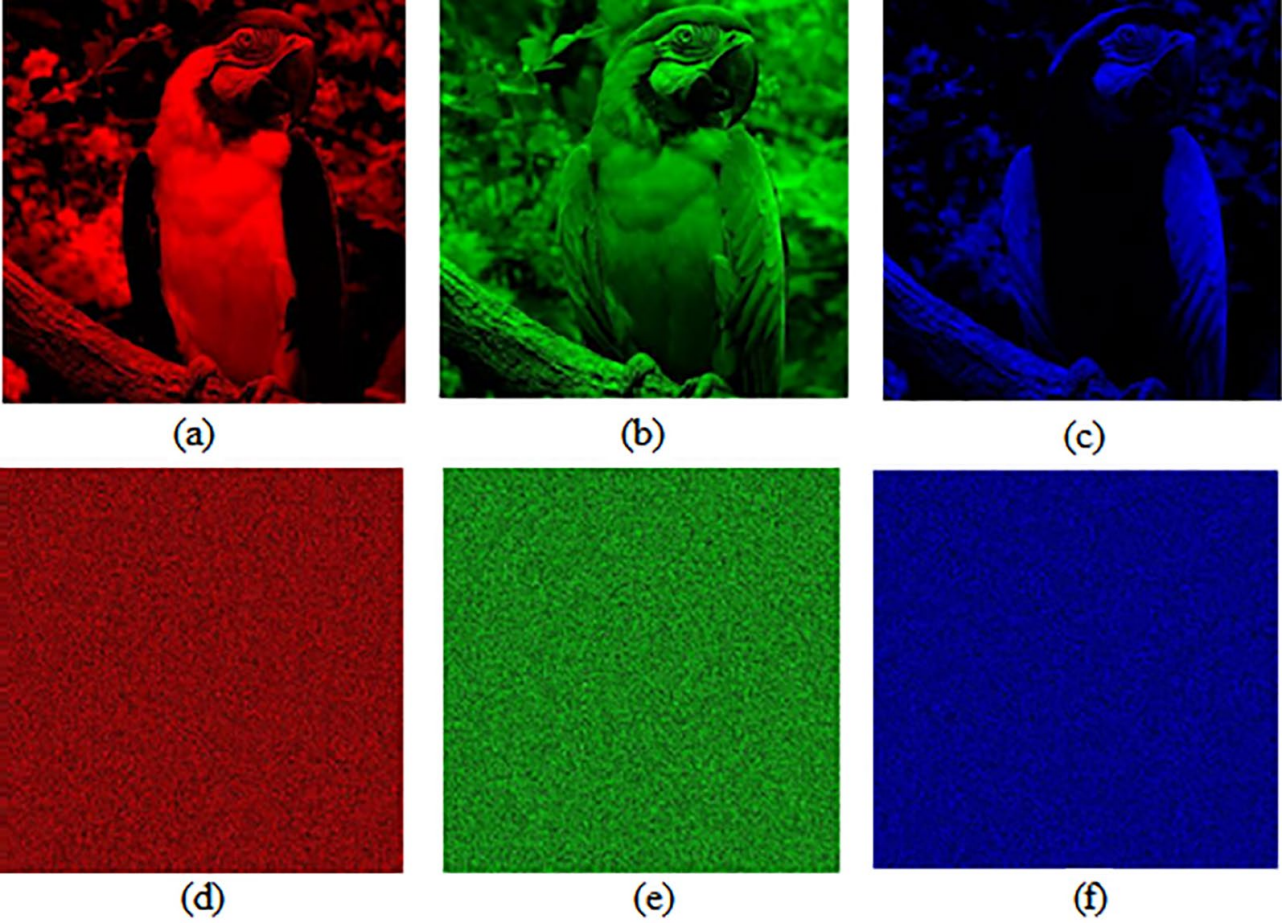
Plain and encrypted images of parrot. (**a**) R channel; (**b**) G channel; (**c**) B channel; (**d**) Encrypted R channel; (**e**) Encrypted G channel; (**f**) Encrypted B channel.

**Figure 8:**
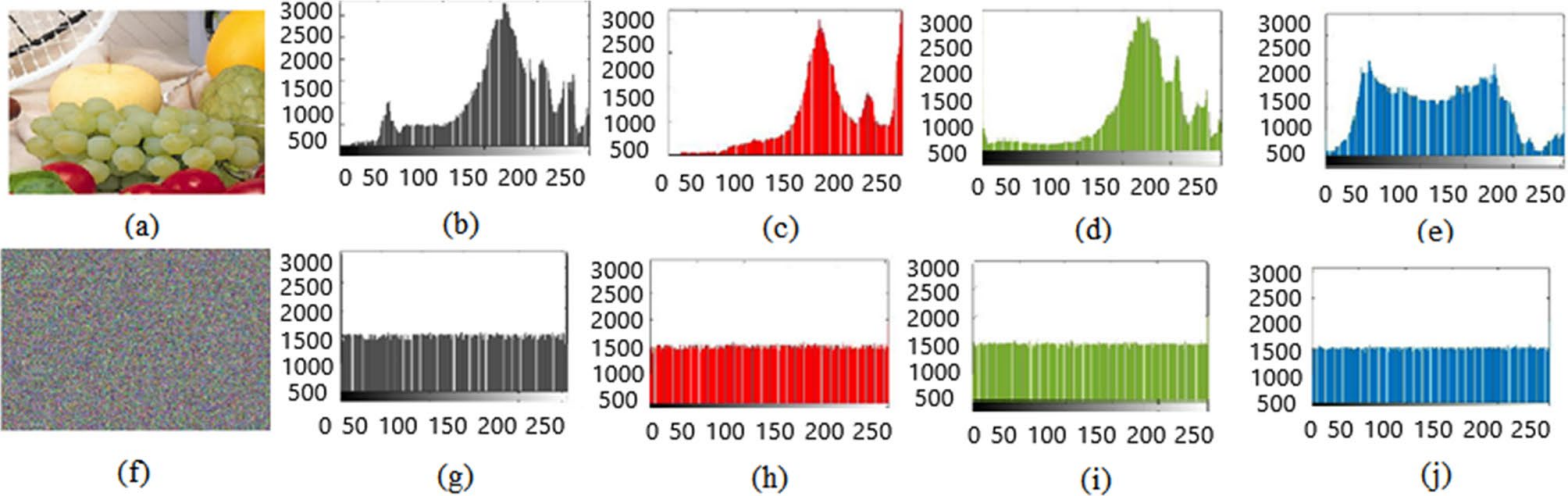
Plain and encrypted images of fruit. (**a**) Color image; (**b**) Histogram of color image; (**c**) Plain R channel; (**d**) Plain G channel; (**e**) Plain B channel; (**f**) Encrypted color image; (**g**) Histogram of encrypted image; (**h**) Encrypted R channel; (**i**) Encrypted G channel; (**j**) Encrypted B channel.

**Figure 9:**
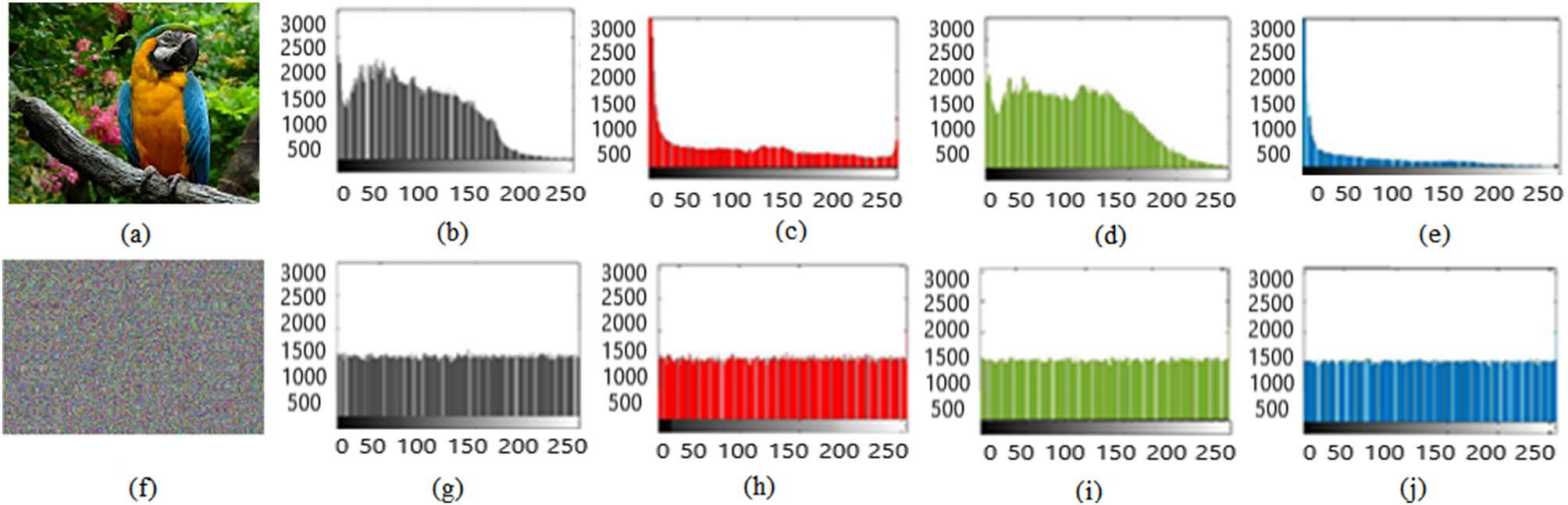
Plain and encrypted images of parrot. (**a**) Color image; (**b**) Histogram of color image; (**c**) Plain R channel; (**d**) Plain G channel; (**e**) Plain B channel; (**f**) Encrypted color image; (**g**). Histogram of encrypted image; (**h**) Encrypted R channel; (**i**) Encrypted G channel; (**j**) Encrypted B channel.

Test images of fruit and parrot with their corresponding encrypted images are shown in Figs. 6 and 7 respectively. Two-dimensional histogram of the plain test images (fruit Fig. 8g, parrot Fig. 9g) over RGB channels are shown in Figs. 8c–e and 9c–e and their corresponding encrypted images histograms are shown in Figs. 8h–j and 9h–j respectively. Histogram of the test images (Figs. 8a and 9a) and their corresponding encrypted images histograms are shown in Figs. 8b,f and 9b, f respectively.

**Figure 10:**
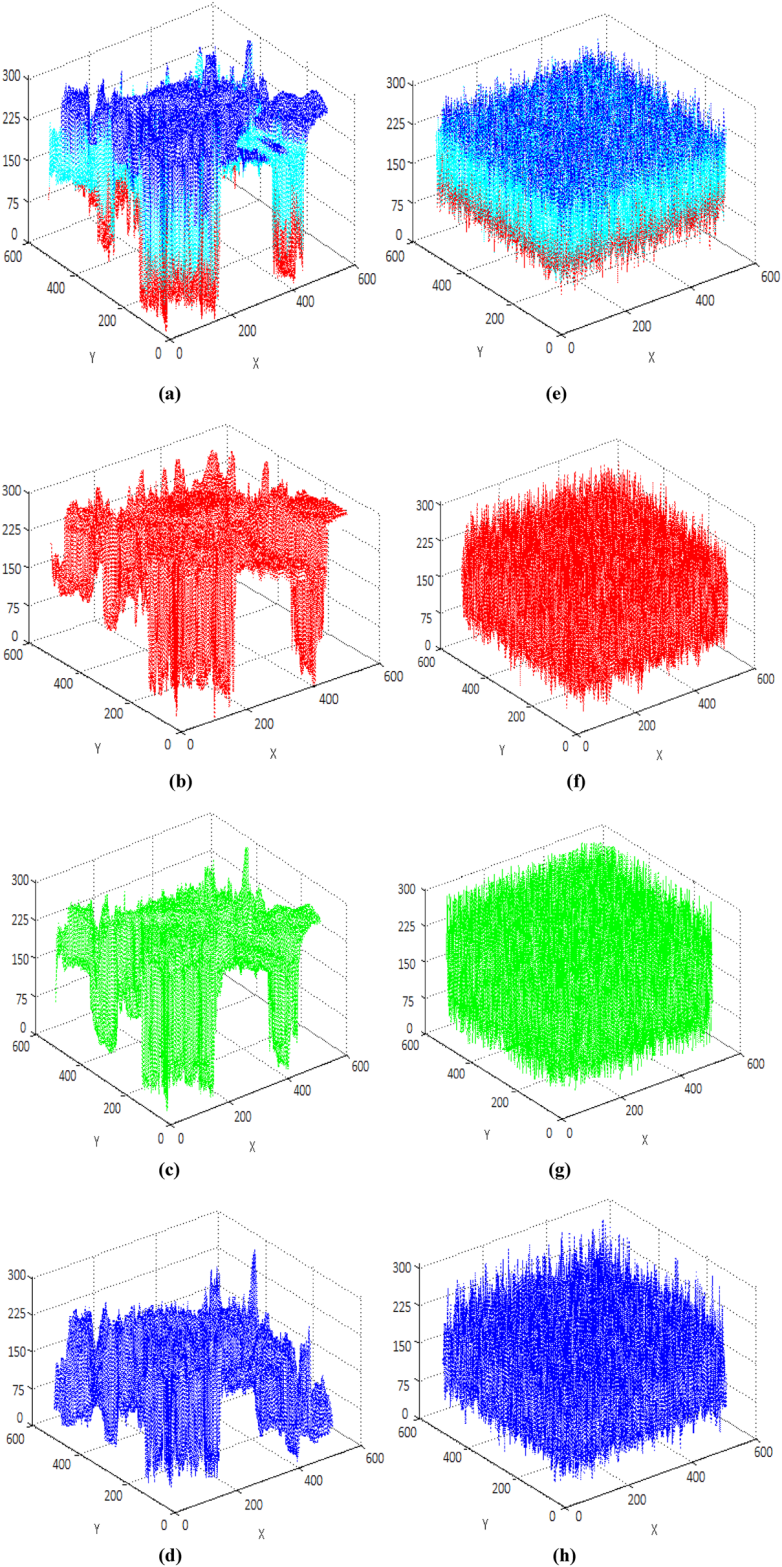
Three-dimensional histogram of fruit. (**a**) Color plain image; (**b**) R channel of plain image; (**c**) G channel of plain image; (**d**) B channel of plain image; (**e**) Color encrypted image; (**f**) R channel of encrypted image; (**g**) G channel of encrypted image; (**h**) B channel of encrypted image.

**Figure 11:**
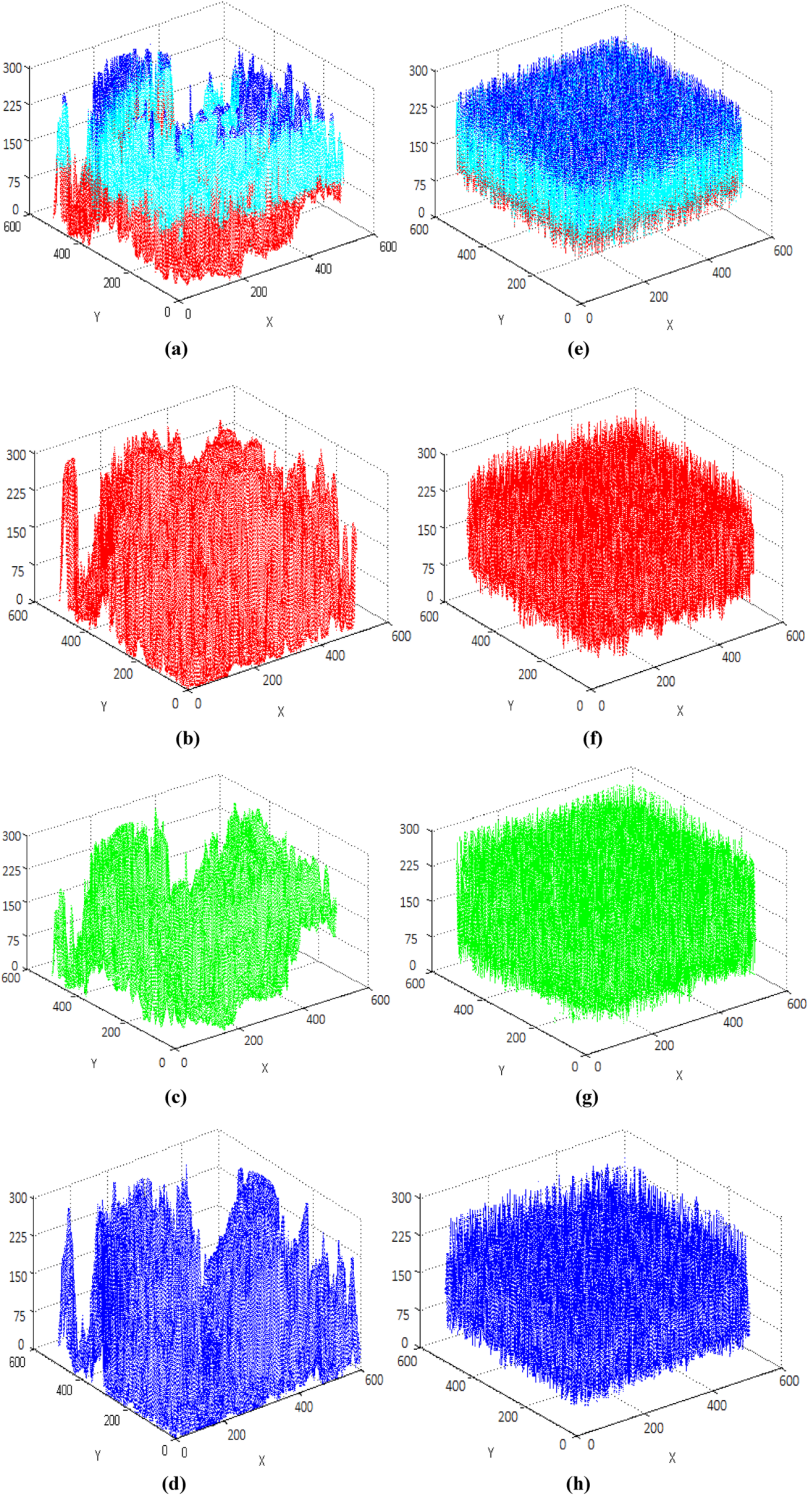
Three-dimensional histogram of parrot. (**a**) Color plain image; (**b**) R channel of plain image; (**c**) G channel of plain image; (**d**) B channel of plain image; (**e**) Color encrypted image; (**f**) R channel of encrypted image; (**g**) G channel of encrypted image; (**h**) B channel of encrypted image.

Three-dimensional histogram of the plain test images (fruit, parrot) over RGB channels are shown in Figs. 10b–d and 11b–d and their corresponding encrypted images histograms are shown in Figs. 10f–h and 11f–h respectively. Histogram of the color plain test images (fruit, parrot) are shown in Figs. 10a and 11a and their corresponding encrypted images histograms are shown in Figs. 10e and 11e respectively.

It is clearly visible that the proposed algorithm shows a uniform two dimensional and 3-dimensional histogram for encrypted image. The uniformity of random pixels indicates a good encryption and resist against several types of statistical attacks. So, histogram analysis fails to provide any clue about encrypted image.

### Correlation-coefficient analysis

**Figure 12:**
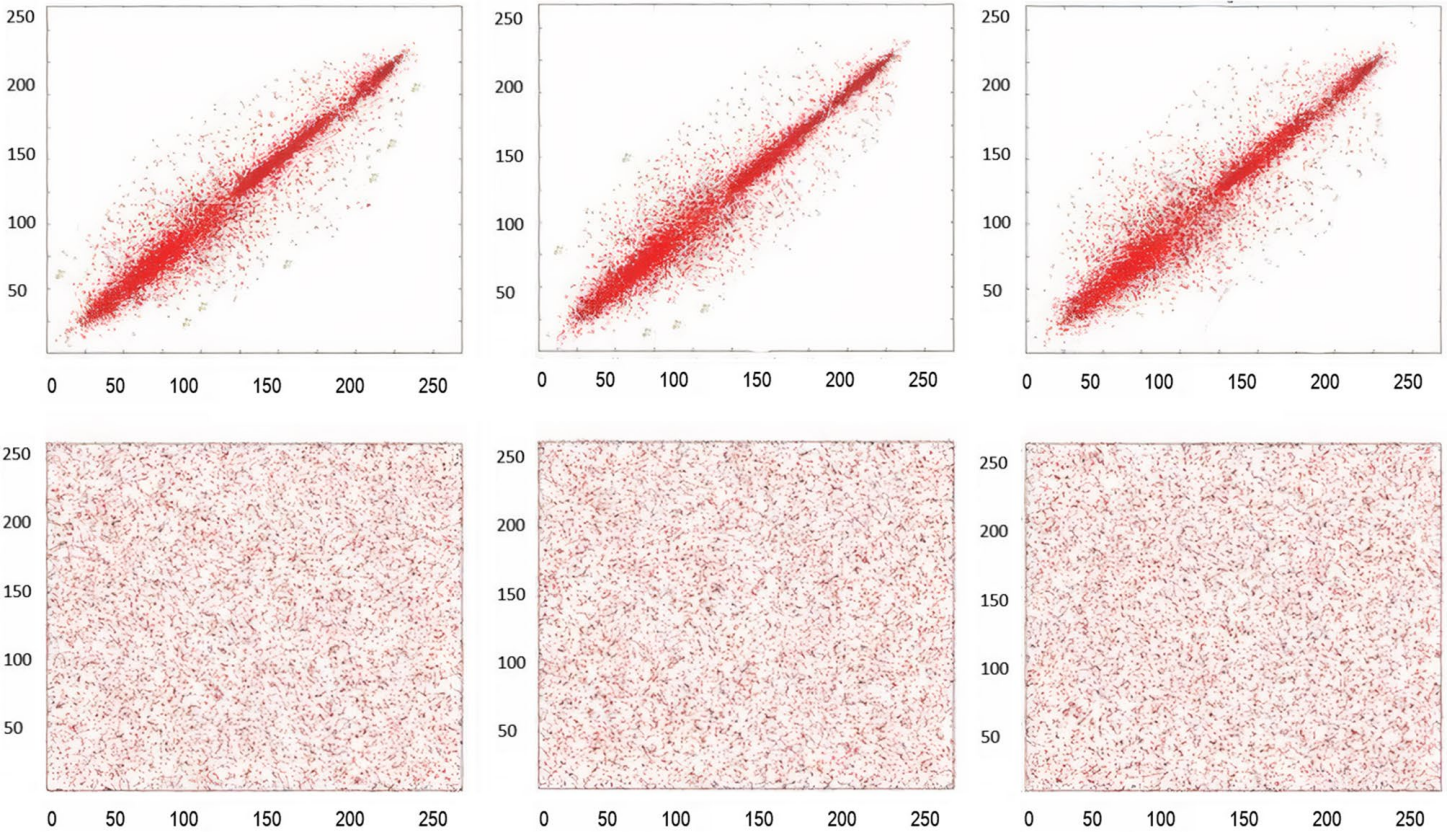
Red channel scatter plots of the Sailboat, to indicate the correlation-coefficient analysis of neighbouring pixels. (**a**) Plain image horizontal direction; (**b**) Plain image vertical direction; (**c**) Plain image diagonal direction; (**d**) Encrypted image horizontal direction; (**e**) Encrypted image vertical direction; (**f**) Encrypted image diagonal direction.

**Figure 13:**
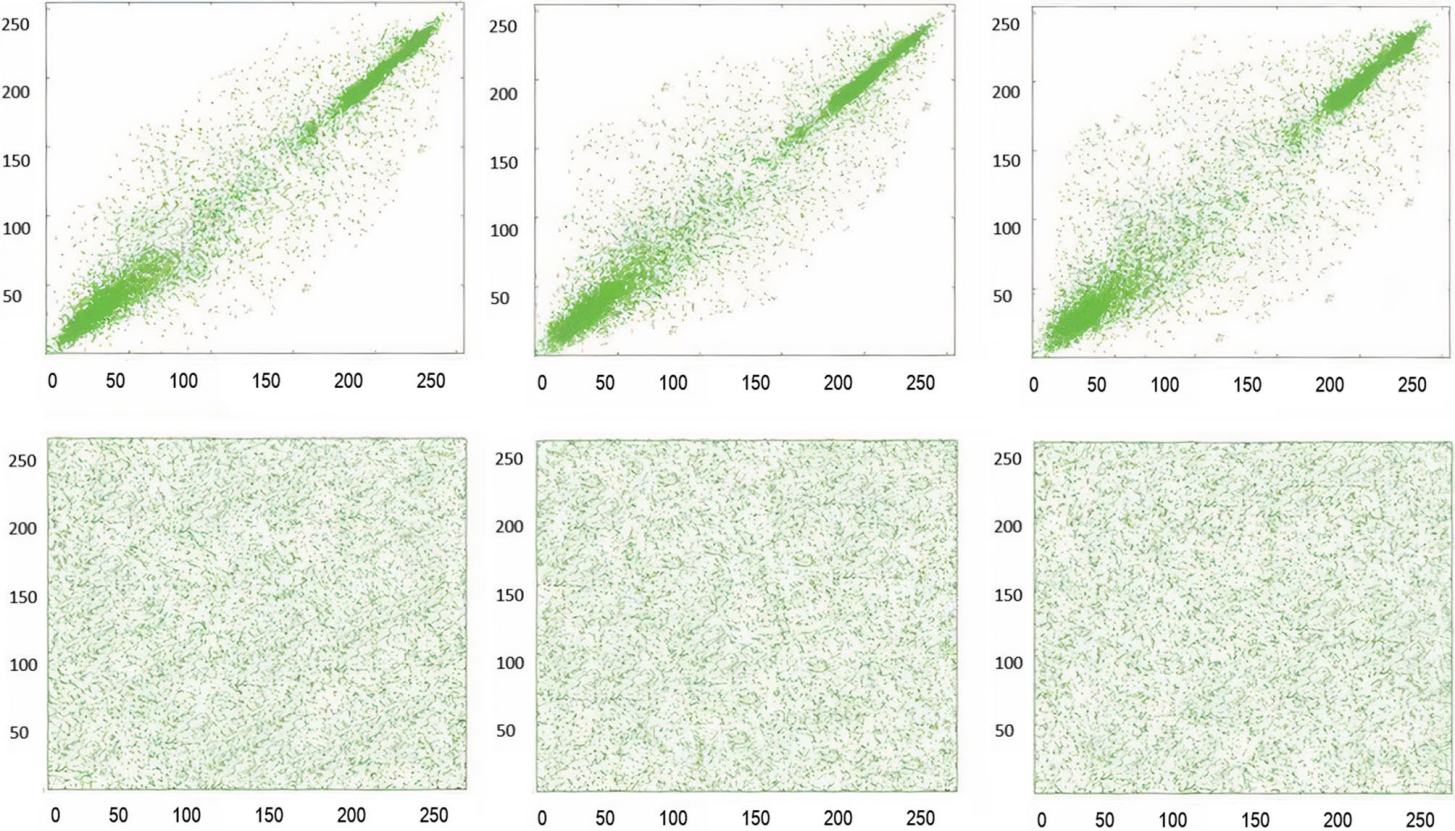
Green channel scatter plots of the Sailboat, to indicate the correlation-coefficient analysis of neighbouring pixels. (**a**) Plain image horizontal direction; (**b**) Plain image vertical direction; (**c**) Plain image diagonal direction; (**d**) Encrypted image horizontal direction; (**e**) Encrypted image vertical direction; (**f**) Encrypted image diagonal direction.

**Figure 14:**
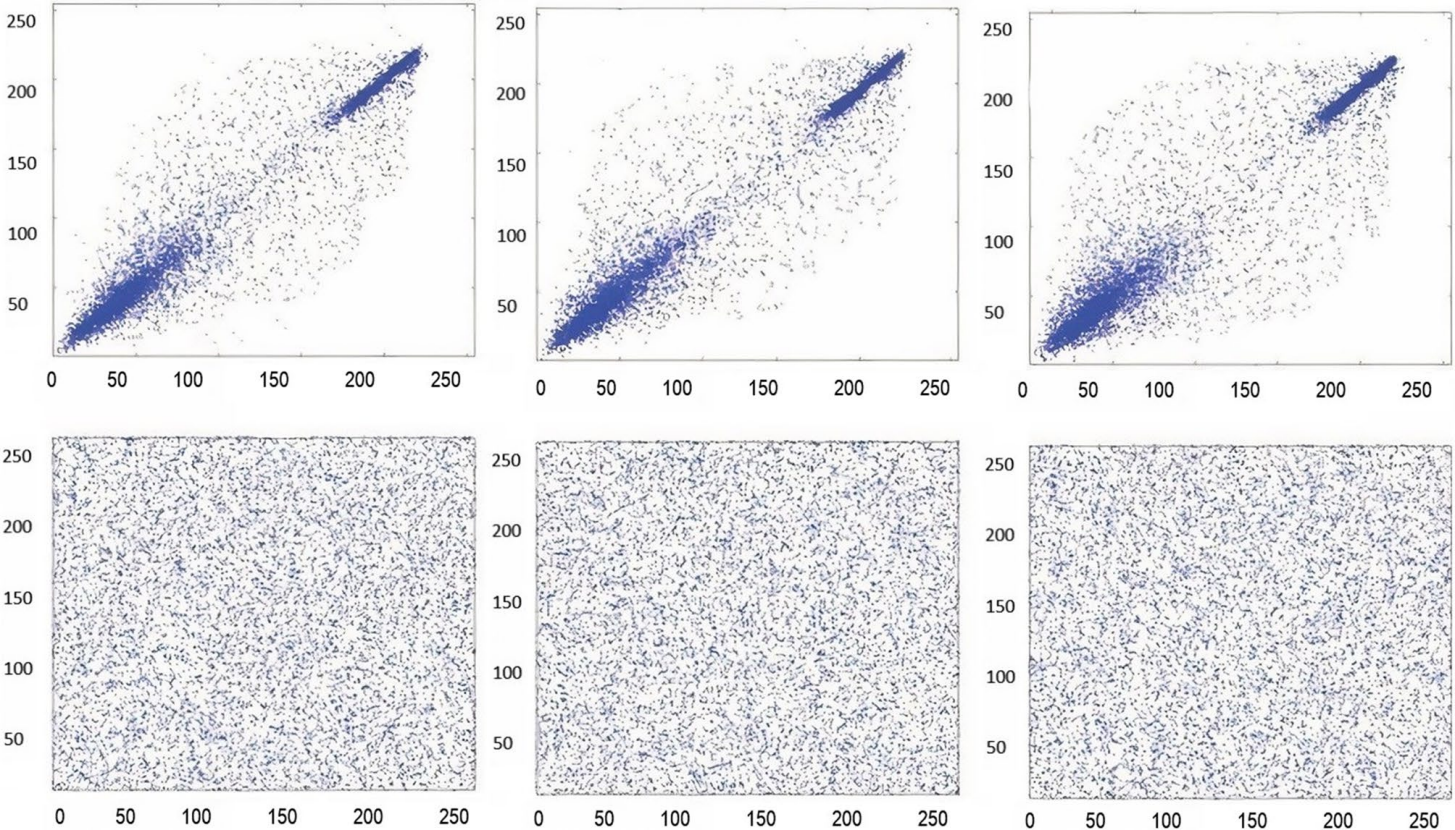
Blue channel scatter plots of the Sailboat, to indicate the correlation-coefficient analysis of neighbouring pixels. (**a**) Plain image horizontal direction; (**b**) Plain image vertical direction; (**c**) Plain image diagonal direction; (**d**) Encrypted image horizontal direction; (**e**) Encrypted image vertical direction; (**f**) Encrypted image diagonal direction.

Adjacent pixels of the plain images are highly correlated, that provides significant visual traits to the attacker. Strong cipher should reduce the correlation of adjacent pixels. Adjacent pixel pairs in every direction of the sailboat image are plotted in Figs. 12, 13, and 14 From the plain images and from the encrypted images, 10^3^ adjacent pixels are selected in the horizontal, vertical and diagonal directions to calculate their correlation coefficients by using Eqs. (9), (10), (11). The results of the Correlation-coefficient analysis for each channel (R, G, B) of Peppers, parrot, baboon, and sailboat are presented in Table 8. It is clear that the distribution of adjacent pixel pairs in every direction and in every channel (R, G, B) are entirely changed after the encryption. Correlation coefficients are calculated by the following formulas:

9 $$ r_{{xy}}  = cov\left( {x,y} \right)/\left( {\sqrt {\psi \left( x \right)} \sqrt {\psi \left( y \right)} } \right. $$

10 $$ cov\left( {x,y} \right) = \frac{1}{N}\mathop \sum \limits_{{i = 1}}^{N} \left( {x_{i}  - E\left( x \right)} \right)\left( {y_{i}  - E\left( y \right)} \right) $$

11 $$ \psi \left( x \right) = \frac{1}{N}\mathop \sum \limits_{{i = 1}}^{N} \left( {x_{i}  - E\left( x \right)} \right)^{2} ,~E\left( x \right) = \frac{1}{N}\mathop \sum \limits_{{i = 1}}^{N} x_{i} $$

**Table 8:** Correlation-coefficient analysis.

Images	Horizontal			Vertical			Diagonal		
	R	G	B	R	G	B	R	G	B
Plain Peppers	0.9853	0.9931	0.9819	0.9788	0.9862	0.9854	0.9866	0.9754	0.9767
Encrypted Peppers	− 0.0210	− 0.0054	− 0.0003	− 0.0114	− 0.0115	− 0.0201	− 0.0037	− 0.0018	− 0.0043
Plain Parrot	0.9659	0.9468	0.9546	0.9402	0.9303	0.9753	0.9784	0.9010	0.9182
Encrypted Parrot	− 0.0016	− 0.0072	− 0.0069	0.0002	− 0.0015	0.0007	− 0.0008	0.0014	0.0006
Plain Baboon	0.9195	0.8519	0.9325	0.8567	0.8982	0.8413	0.8478	0.8886	0.8689
Encrypted Baboon	− 0.0069	− 0.0143	− 0.0048	− 0.0203	− 0.0187	− 0.0032	− 0.0019	− 0.0164	− 0.0053
Plain Sailboat	0.9548	0.9552	0.9637	0.9573	0.9627	0.9507	0.9322	0.9147	0.9246
Encrypted Sailboat	− 0.0002	− 0.0084	− 0.001	− 0.0019	− 0.0018	0.0038	0.0009	0.005	0.0011

where x, y shows 2 adjacent pixels (whatever diagonal, vertical, or horizontal), N is the total size of $x_{i}$ and $y_{i}$ acquired form the image. $E\left( x \right)$ is the mean value of $x_{i}$ and, $E\left( y \right)\;is\;the\;mean\;value\;of$ $y_{i}$.

Results presented in Table 8 show that before encryption, correlation-coefficient values of plain images are close to 1 and after encryption, these values are close to 0 which validates that correlation-coefficient analysis fails to provide any clue of plain images.

Assume that our proposed S-box and any existing s-box (such as the AES) are identical, even in this case our proposed S-box does not hold those vulnerabilities which are highlighted in above "Weaknesses in existing S-box designs" section. Instead of well-understood mathematical principles, we introduce the true random numbers for the design of S-boxes due to the reason that, true random numbers are irreversible, unpredictable, and unreproducible, even if their internal structure and response history are known to adversaries.

The asymptotic computational complexity of our technique is O(n^2^), detailed derivation is attached in Annexed C. The asymptotic computational complexity of^1^ and^75^ is O(n^4^) and O(n^5^) respectively. The asymptotic computational complexity of^125–127^ is O(n^3^).

## Conclusion

Protecting confidential data is a major worldwide challenge and block ciphers has been a standout amongst the most reliable option by which data security is accomplished. Block cipher strength against various attacks rely on substitution box. Various weakness in the algebraic and chaos-based S-box designs are pointed in the literature. On the other hand, researchers endorse the true random sequences for cryptography due to the fact that these sequences rely on the strength of naturally occurring processes to generate the true randomness. The objective of this research is extraction of inevitable random noise from the medical imaging, and to synthesize the multi-level information fusion for the construction of strong substitution boxes. Various security evaluation criteria, statistical tests and comparison with various very recent state-of-the-art techniques validate our proposed technique. In future, we will extend our proposed information fusion design for the construction of lattice cryptographic primitive.

## Supplementary Information

Supplementary Information 1.

Supplementary Information 2.

Supplementary Information 3.
