# Supplementary material for: Multilevel information fusion for cryptographic substitution box construction based on inevitable random noise in medical imaging
Source: Sci Rep. 2021 Jul 12;11:14282. doi: 10.1038/s41598-021-93344-z (PMC8275796; doi:10.1038/s41598-021-93344-z)
Supplement: Supplementary file 1 — Supplementary Information 1. [file 41598_2021_93344_MOESM1_ESM.pdf]

Multilevel Information Fusion for Cryptographic Substitution Box Construction based on Inevitable Random Noise in Medical Imaging

Muhammad Fahad Khan [1, 2], Khalid Saleem [1], Mohammed Ali Alshara [3], Shariq Bashir [4]

[1] Department of Computer Sciences, Quaid-i-Azam University, Islamabad,
[2] Department of Software Engineering, Foundation University Islamabad, Pakistan
[3] Department of Information Technology, College of Computer and Information Sciences, Imam Mohammad Ibn Saud Islamic University, Riyadh, Saudi Arabia
[4] The College of Arts and Sciences, DMPS Computer Science Section, University of Nizwa, Sultanate of Oman

Correspondence to: **mfkhan@cs.qau.edu.pk**,**fahad.khan@fui.edu.pk**

………

Step-by-step example for the calculation of 1st element of the Sbox-1

X= T_nf: [10011100, 01110000, 10001000, 11000001, 10011001, 11100101, 00001011, 00011001, 00111110, 00100000, 01001010, 10000010]

Z= Sp_nf: [00001011, 10110111, 00101101, 11011010, 11111101, 00111010, 10001000, 00010110, 01111001, 01000010, 00100000, 00111010]

Y= Sh_nf: [00000100, 01101011, 01000110, 00111010, 10111100, 10110001, 10111011, 10101001, 00001110, 10000001, 10000000, 10100001]

W= Ip_nf: [11110100, 01101011, 00010001, 11101111, 01011000, 11110001, 00101100, 01000100, 00100110, 00111000, 11100101, 11000001]

Step 1: Binary Form

T_nf: [10011100, 01110000, 10001000]

Sp_nf: [00001011, 10110111, 00101101]

Sh_nf: [00000100, 01101011, 01000110]

Ip_nf: [11110100, 01101011, 00010001]

Horizontal permutation row: 0

Step 2: Checking LSB of10011100

Step 4:Frequeny of 0's x<y so permutate(y,w,x,z)

10011100 00001010 00000100 11110101

Horizontal permutation row: 1

Step 2: Checking LSB of01110000

Step 3:Frequeny of 0's x>y so permutate(x,z,y,w)

01110001 10110111 01101010 01101011

Horizontal permutation row: 2

Step 2: Checking LSB of10001000

Step 3:Frequeny of 0's x>y so permutate(x,z,y,w)

10001000 00101101 01000110 00010001

Vertical Permutation:

Step 1: 11001000

Step 2: 6bit number50

Step 3 First bit of octet is 0: permutate column x

[10011101, 01110000, 10001000]

Step 5 2nd bit of octet is 0: Permutate column z

[00001011, 10110111, 00101100]

Substitution boxes construction (L3): step 1

Th: [10011101, 01110000, 10001000]

Sp: [00001011, 10110111, 00101100]

Sh: [00000100, 01101010, 01000110]

Ip: [11110101, 01101011, 00010001]

final: [10011101, 00001011, 00000100, 11110101, 01110000, 10110111, 01101010, 01101011, 10001000, 00101100, 01000110, 00010001]

final: [157, 11, 4, 245, 112, 183, 106, 107, 136, 44, 70, 17]

Step 2 Block: 0

[157, 11, 4, 245, 112, 183]

Step 3: 100111010000101100000100111101010111000010110111

100 111 010 000 101 100 000 100 111 101 010 111 000 010 110 111

Step 4: Frequency of 0: [2, 0, 2, 3, 1, 2, 3, 2, 0, 1, 2, 0, 3, 2, 1, 0]

Step 5 to 8 Map1 filling

[[2, 0, 2, 3], [1, 2, 3, 2], [0, 1, 2, 0], [3, 2, 1, 0]]

Step 9 Map2 filling

[[2, 0, 2, 2], [0, 1, 3, 0], [1, 3, 3, 1], [2, 2, 2, 0]]

Step 10 11 : [2, 0, 1, 3]

data[157, 11, 4, 245]

Step 12 13 14: [0, 0, 3]

Step 15 16 17: 4, 11, 157,

Step 18 19: 1,3

Step 20: 2 and 2 passed to Map1: 157

Step 21: 2

Step 22: binary of 6th right index: 100111

Step 23: Binary with LSB & MSB: 10011100

Step 24: decimal number = 156 (**σ**)

Step 1: Binary Form

T_nf: [11000001, 10011001, 11100101]

Sp_nf: [11011010, 11111101, 00111010]

Sh_nf: [00111010, 10111100, 10110001]

Ip_nf: [11101111, 01011000, 11110001]

Horizontal permutation row: 0

Step 2: Checking LSB of11000001

Step 10:Frequeny of 1's x<y so permutate(y,w,x,z)

11000000 11011011 00111011 11101110

Horizontal permutation row: 1

Step 2: Checking LSB of10011001

Step 10:Frequeny of 1's x<y so permutate(y,w,x,z)

10011000 11111100 10111101 01011001

Horizontal permutation row: 2

Step 2: Checking LSB of11100101

Step 11: same frequency of x & y

Step 12:Frequeny of 1's z>w so permutate(z,y,w,x)

11100101 00111011 10110001 11110000

Vertical Permutation:

Step 1: 01000101

Step 2: 6bit number17

Step 4 First bit of octet is 1: permutate column y

[00111011, 10111101, 10110001]

Step 5 2nd bit of octet is 0: Permutate column z

[11011010, 11111101, 00111011]

Substitution boxes construction (L3): step 1

Th: [11000000, 10011000, 11100101]

Sp: [11011010, 11111101, 00111011]

Sh: [00111011, 10111101, 10110001]

Ip: [11101110, 01011001, 11110000]

final: [11000000, 11011010, 00111011, 11101110, 10011000, 11111101, 10111101, 01011001, 11100101, 00111011, 10110001, 11110000]

final: [192, 218, 59, 238, 152, 253, 189, 89, 229, 59, 177, 240]

Step 2 Block: 0

[192, 218, 59, 238, 152, 253]

Step 3: 110000001101101000111011111011101001100011111101

110 000 001 101 101 000 111 011 111 011 101 001 100 011 111 101

Step 4: Frequency of 0: [1, 3, 2, 1, 1, 3, 0, 1, 0, 1, 1, 2, 2, 1, 0, 1]

Step 5 to 8 Map1 filling

[[1, 3, 2, 1], [1, 3, 0, 1], [0, 1, 1, 2], [2, 1, 0, 1]]

Step 9 Map2 filling

[[1, 0, 2, 1], [3, 1, 1, 2], [1, 2, 0, 0], [3, 1, 1, 1]]

Step 10 11 : [1, 0, 2, 3]

data[192, 218, 59, 238]

Step 12 13 14: [1, 0, 1]

Step 15 16 17: 238, 192, 192,

Step 18 19: 1,3

Step 20: 1 and 1 passed to Map1: 238

Step 21: 3

Step 22: binary of 6th right index: 111001

Step 23: Binary with LSB & MSB: 11100110

Step 24: decimal number = 230 (**β**)

Step 1: Binary Form

T_nf: [00001011, 00011001, 00111110]

Sp_nf: [10001000, 00010110, 01111001]

Sh_nf: [10111011, 10101001, 00001110]

Ip_nf: [00101100, 01000100, 00100110]

Horizontal permutation row: 0

Step 2: Checking LSB of00001011

Step 10:Frequeny of 1's x<y so permutate(y,w,x,z)

00001011 10001000 10111011 00101100

Horizontal permutation row: 1

Step 2: Checking LSB of00011001

Step 11: same frequency of x & y

Step 12:Frequeny of 1's z>w so permutate(z,y,w,x)

00011001 00010110 10101001 01000100

Horizontal permutation row: 2

Step 2: Checking LSB of00111110

Step 4:Frequeny of 0's x<y so permutate(y,w,x,z)

00111110 01111000 00001110 00100111

Vertical Permutation:

Step 1: 10000001

Step 2: 6bit number32

Step 4 First bit of octet is 1: permutate column y

[10111010, 10101001, 00001111]

Step 5 2nd bit of octet is 0: Permutate column z

[10001000, 00010110, 01111000]

Substitution boxes construction (L3): step 1

Th: [00001011, 00011001, 00111110]

Sp: [10001000, 00010110, 01111000]

Sh: [10111010, 10101001, 00001111]

Ip: [00101100, 01000100, 00100111]

final: [00001011, 10001000, 10111010, 00101100, 00011001, 00010110, 10101001, 01000100, 00111110, 01111000, 00001111, 00100111]

final: [11, 136, 186, 44, 25, 22, 169, 68, 62, 120, 15, 39]

Step 2 Block: 0

[11, 136, 186, 44, 25, 22]

Step 3: 000010111000100010111010001011000001100100010110

000 010 111 000 100 010 111 010 001 011 000 001
100 100 010 110

Step 4: Frequency of 0: [3, 2, 0, 3, 2, 2, 0, 2, 2, 1, 3, 2, 2, 2, 2, 1]

Step 5 to 8 Map1 filling

[[3, 2, 0, 3], [2, 2, 0, 2], [2, 1, 3, 2], [2, 2, 2, 1]]

Step 9 Map2 filling

[[3, 2, 0, 3], [2, 1, 3, 2], [2, 2, 0, 2], [2, 2, 2, 1]]

Step 10 11 : [3, 2, 0, 1]

data[11, 136, 186, 44]

Step 12 13 14: [2, 2, 1]

Step 15 16 17: 136, 136, 136,

Step 18 19: 3,2

Step 20: 2 and 2 passed to Map1: 11

Step 21: 3

Step 22: binary of 6th right index: 000101

Step 23: Binary with LSB & MSB: 00010110

Step 24: decimal number = 22 **(γ)**

Step 1: Binary Form

T_nf: [00100000, 01001010, 10000010]

Sp_nf: [01000010, 00100000, 00111010]

Sh_nf: [10000001, 10000000, 10100001]

Ip_nf: [00111000, 11100101, 11000001]

Horizontal permutation row: 0

Step 2: Checking LSB of00100000

Step 5: same frequency of 0's in x & y

Step 6:Frequeny of 0's z>w so permutate(z,y,w,x)

00100001 01000010 10000000 00111000

Horizontal permutation row: 1

Step 2: Checking LSB of01001010

Step 4:Frequeny of 0's x<y so permutate(y,w,x,z)

01001010 00100001 10000000 11100100

Horizontal permutation row: 2

Step 2: Checking LSB of10000010

Step 3:Frequeny of 0's x>y so permutate(x,z,y,w)

10000011 00111011 10100000 11000000

Vertical Permutation:

Step 1: 01001101

Step 2: 6bit number19

Step 4 First bit of octet is 1: permutate column y

[10000000, 10000000, 10100000]

Step 5 2nd bit of octet is 0: Permutate column z

[01000011, 00100001, 00111010]

Substitution boxes construction (L3): step 1

Th: [00100001, 01001010, 10000011]

Sp: [01000011, 00100001, 00111010]

Sh: [10000000, 10000000, 10100000]

Ip: [00111000, 11100100, 11000000]

final: [00100001, 01000011, 10000000, 00111000, 01001010, 00100001, 10000000, 11100100, 10000011, 00111010, 10100000, 11000000]

final: [33, 67, 128, 56, 74, 33, 128, 228, 131, 58, 160, 192]

Step 2 Block: 0

[33, 67, 128, 56, 74, 33]

Step 3: 001000010100001110000000001110000100101000100001

001 000 010 100 001 110 000 000 001 110 000 100 101 000 100 001

Step 4: Frequency of 0: [2, 3, 2, 2, 2, 1, 3, 3, 2, 1, 3, 2, 1, 3, 2, 2]

Step 5 to 8 Map1 filling

[[2, 3, 2, 2], [2, 1, 3, 3], [2, 1, 3, 2], [1, 3, 2, 2]]

Step 9 Map2 filling

[[2, 2, 2, 3], [3, 1, 2, 2], [2, 1, 3, 2], [1, 3, 3, 2]]

Step 10 11 : [2, 3, 1, 0]

data[33, 67, 128, 56]

Step 12 13 14: [2, 1, 2]

Step 15 16 17: 33, 128, 67,

Step 18 19: 1,3

Step 20: 3 and 3 passed to Map1: 33

Step 21: 2

Step 22: binary of 6th right index: 000000

Step 23: Binary with LSB & MSB: 00000001

Step 24: decimal number = 1 (**δ**)

Put the values of σ, β, γ and δ for z = 0

f (z_i) ↦(σ_i z_i + β_i)/(γ_i z_i+ δ1)

156*0 + 230 / 22*0 + 1

230/1

230

First value of the S-box is 230
