# Supplementary material for: Multilevel information fusion for cryptographic substitution box construction based on inevitable random noise in medical imaging
Source: Sci Rep. 2021 Jul 12;11:14282. doi: 10.1038/s41598-021-93344-z (PMC8275796; doi:10.1038/s41598-021-93344-z)
Supplement: Supplementary file 2 — Supplementary Information 2. [file 41598_2021_93344_MOESM2_ESM.pdf]

Multilevel Information Fusion for Cryptographic Substitution Box Construction based on Inevitable Random Noise in Medical Imaging

Muhammad Fahad Khan [1, 2], Khalid Saleem [1], Mohammed Ali Alshara [3], Shariq Bashir [4]

[1] Department of Computer Sciences, Quaid-i-Azam University, Islamabad,
[2] Department of Software Engineering, Foundation University Islamabad, Pakistan
[3] Department of Information Technology, College of Computer and Information Sciences, Imam Mohammad Ibn Saud Islamic University, Riyadh, Saudi Arabia
[4] The College of Arts and Sciences, DMPS Computer Science Section, University of Nizwa, Sultanate of Oman
Correspondence to: **mfkhan@cs.qau.edu.pk**,**fahad.khan@fui.edu.pk**

……

Step-by-step example for the calculation of 1st element of the Sbox-2

X= T_nf: [00010100, 11111010, 01101110, 11001101, 01010110, 01000111, 01101101, 10000110, 01100011, 00010101, 01110000, 10011010]

Z= Sp_nf: [00010100, 01100000, 10101010, 11101001, 10000010, 10010001, 10111110, 11111100, 10101001, 01101000, 11010000, 01110111]

Y= Sh_nf: [10111001, 00100111, 11010010, 01111010, 11110101, 11000000, 11000011, 00101010, 11100110, 11100001, 01011011, 00101000]

W= Ip_nf: [00110111, 01110000, 11111111, 11111111, 00010110, 01001011, 00100110, 01011001, 00100100, 10110011, 00110011, 10111111]

Step 1: Binary Form

T_nf: [00010100, 11111010, 01101110]

Sp_nf: [00010100, 01100000, 11010010]

Sh_nf: [10111001, 00100111, 11010010]

Ip_nf: [00110111, 01110000, 11111111]

Horizontal permutation row: 0

Step 2: Checking LSB of00010100

Step 3:Frequeny of 0's x>y so permutate(x,z,y,w)

00010101 00010101 10111000 00110110

Horizontal permutation row: 1

Step 2: Checking LSB of11111010

Step 5: same frequency of 0's in x & y

Step 6: Frequency of 0's z>w so permutate(z,y,w,x)

11111011 01100000 00100110 01110000

Horizontal permutation row: 2

Step 2: Checking LSB of01101110

Step 4:Frequeny of 0's x<y so permutate(y,w,x,z)

01101110 11010011 11010010 11111110

Vertical Permutation:

Step 1: 01111000

Step 2: 6bit number30

Step 3 First bit of octet is 0: permutate column x

[00010101, 11111010, 01101111]

Step 5 2nd bit of octet is 0: Permutate column z

[00010100, 01100001, 11010011]

Substitution boxes construction (L3): step 1

Th: [00010101, 11111010, 01101111]

Sp: [00010100, 01100001, 11010011]

Sh: [10111000, 00100110, 11010010]

Ip: [00110110, 01110000, 11111110]

final: [00010101, 00010100, 10111000, 00110110, 11111010, 01100001, 00100110, 01110000, 01101111, 11010011, 11010010, 11111110]

final: [21, 20, 184, 54, 250, 97, 38, 112, 111, 211, 210, 254]

Step 2 Block: 0

[21, 20, 184, 54, 250, 97]

Step 3: 000101010001010010111000001101101111101001100001

000 101 010 001 010 010 111 000 001 101 101 111 101 001 100 001

Step 4: Frequency of 0: [3, 1, 2, 2, 2, 2, 0, 3, 2, 1, 1, 0, 1, 2, 2, 2]

Step 5 to 8 Map1 filling

[[3, 1, 2, 2], [2, 2, 0, 3], [2, 1, 1, 0], [1, 2, 2, 2]]

Step 9 Map2 filling

[[3, 2, 2, 1], [1, 1, 2, 0], [2, 1, 0, 2], [2, 2, 3, 2]]

Step 10 11 : [3, 2, 1, 0]

data[21, 20, 184, 54]

Step 12 13 14: [2, 1, 2]

Step 15 16 17: 21, 184, 184,

Step 18 19: 2,1

Step 20: 1 and 1 passed to Map1: 20

Step 21: 2

Step 22: binary of 6th right index: 101001

Step 23: Binary with LSB & MSB: 10100101

Step 24: decimal number = 165 **(σ)**

Step 1: Binary Form

T_nf: [11001101, 01010110, 01000111]

Sp_nf: [11101001, 10000010, 10010001]

Sh_nf: [01111010, 11110101, 11000000]

Ip_nf: [11111111, 00010110, 01001011]

Horizontal permutation row: 0

Step 2: Checking LSB of11001101

Step 10: Frequeny of 1's x<y so permutate(y,w,x,z)

11001100 11101001 01111011 11111111

Horizontal permutation row: 1

Step 2: Checking LSB of01010110

Step 3: Frequeny of 0's x>y so permutate(x,z,y,w)

01010111 10000010 11110100 00010110

Horizontal permutation row: 2

Step 2: Checking LSB of01000111

Step 9: Frequeny of 1's x>y so permutate(x,z,y,w)

01000110 10010001 11000001 01001011

Vertical Permutation:

Step 1: 11011011

Step 2: 6bit number54

Step 4 First bit of octet is 1: permutate column y

[01111011, 11110101, 11000000]

Step 6 2nd bit of octet is 1: Permutate column w

[11111111, 00010111, 01001010]

Substitution boxes construction (L3): step 1

Th: [11001100, 01010111, 01000110]

Sp: [11101001, 10000010, 10010001]

Sh: [01111011, 11110101, 11000000]

Ip: [11111111, 00010111, 01001010]

final: [11001100, 11101001, 01111011, 11111111, 01010111, 10000010, 11110101, 00010111, 01000110, 10010001, 11000000, 01001010]

final: [204, 233, 123, 255, 87, 130, 245, 23, 70, 145, 192, 74]

Step 2 Block: 0

[204, 233, 123, 255, 87, 130]

Step 3: 110011001110100101111011111111110101011110000010

110 011 001 110 100 101 111 011 111 111 110 101 011 110 000 010

Step 4: Frequency of 0: [1, 1, 2, 1, 2, 1, 0, 1, 0, 0, 1, 1, 1, 1, 3, 2]

Step 5 to 8 Map1 filling

[[1, 1, 2, 1], [2, 1, 0, 1], [0, 0, 1, 1], [1, 1, 3, 2]]

Step 9 Map2 filling

[[1, 0, 2, 1], [1, 0, 1, 1], [2, 1, 0, 3], [1, 1, 1, 2]]

Step 10 11 : [1, 0, 2, 3]

data[204, 233, 123, 255]

Step 12 13 14: [2, 1, 1]

Step 15 16 17: 204, 233, 204,

Step 18 19: 2,0

Step 20: 1 and 0 passed to Map1: 123

Step 21: 2

Step 22: binary of 6th right index: 011000

Step 23: Binary with LSB & MSB: 01100010

Step 24: decimal number = 98 **(β)**

Step 1: Binary Form

T_nf: [01101101, 10000110, 01100011]

Sp_nf: [10111110, 11111100, 10101001]

Sh_nf: [11000011, 00101010, 11100110]

Ip_nf: [00100110, 01011001, 00100100]

Horizontal permutation row: 0

Step 2: Checking LSB of01101101

Step 9: Frequeny of 1's x>y so permutate(x,z,y,w)

01101101 10111110 11000011 00100110

Horizontal permutation row: 1

Step 2: Checking LSB of10000110

Step 3:Frequeny of 0's x>y so permutate(x,z,y,w)

10000110 11111101 00101010 01011000

Horizontal permutation row: 2

Step 2: Checking LSB of01100011

Step 11: same frequency of x & y

Step 12:Frequeny of 1's z>w so permutate(z,y,w,x)

01100010 10101000 11100111 00100101

Vertical Permutation:

Step 1: 00100110

Step 2: 6bit number9

Step 3 First bit of octet is 0: permutate column x

[01101100, 10000110, 01100011]

Step 6 2nd bit of octet is 1: Permutate column w

[00100111, 01011000, 00100100]

Substitution boxes construction (L3): step 1

Th: [01101100, 10000110, 01100011]

Sp: [10111110, 11111101, 10101000]

Sh: [11000011, 00101010, 11100111]

Ip: [00100111, 01011000, 00100100]

final: [01101100, 10111110, 11000011, 00100111, 10000110, 11111101, 00101010, 01011000, 01100011, 10101000, 11100111, 00100100]

final: [108, 190, 195, 39, 134, 253, 42, 88, 99, 168, 231, 36]

Step 2 Block: 0

[108, 190, 195, 39, 134, 253]

Step 3: 011011001011111011000011001001111000011011111101

011 011 001 011 111 011 000 011 001 001 111 000 011 011 111 101

Step 4: Frequency of 0: [1, 1, 2, 1, 0, 1, 3, 1, 2, 2, 0, 3, 1, 1, 0, 1]

Step 5 to 8 Map1 filling

[[1, 1, 2, 1], [0, 1, 3, 1], [2, 2, 0, 3], [1, 1, 0, 1]]

Step 9 Map2 filling

[[1, 2, 2, 0], [1, 2, 1, 3], [0, 1, 3, 0], [1, 1, 1, 1]]

Step 10 11 : [1, 2, 0, 3]

data[108, 190, 195, 39]

Step 12 13 14: [3, 1, 1]

Step 15 16 17: 108, 108, 39,

Step 18 19: 1,1

Step 20: 3 and 1 passed to Map1: 108

Step 21: 1

Step 22: binary of 6th right index: 111010

Step 23: Binary with LSB & MSB: 11101000

Step 24: decimal number = 232 **(γ)**

Step 1: Binary Form

T_nf: [00010101, 01110000, 10011010]

Sp_nf: [01101000, 11010000, 01110111]

Sh_nf: [11100001, 01011011, 00101000]

Ip_nf: [10110011, 00110011, 10111111]

Horizontal permutation row: 0

Step 2: Checking LSB of00010101

Step 9:Frequeny of 1's x>y so permutate(x,z,y,w)

00010101 01101001 11100001 10110010

Horizontal permutation row: 1

Step 2: Checking LSB of01110000

Step 3:Frequeny of 0's x>y so permutate(x,z,y,w)

01110001 11010001 01011010 00110010

Horizontal permutation row: 2

Step 2: Checking LSB of10011010

Step 4:Frequeny of 0's x<y so permutate(y,w,x,z)

10011010 01110111 00101000 10111111

Vertical Permutation:

Step 1: 01011110

Step 2: 6bit number23

Step 3 First bit of octet is 0: permutate column x

[00010101, 01110000, 10011011]

Step 6 2nd bit of octet is 1: Permutate column w

[10110011, 00110010, 10111110]

Substitution boxes construction (L3): step 1

Th: [00010101, 01110000, 10011011]

Sp: [01101001, 11010001, 01110111]

Sh: [11100001, 01011010, 00101000]

Ip: [10110011, 00110010, 10111110]

final: [00010101, 01101001, 11100001, 10110011, 01110000, 11010001, 01011010, 00110010, 10011011, 01110111, 00101000, 10111110]

final: [21, 105, 225, 179, 112, 209, 90, 50, 155, 119, 40, 190]

Step 2 Block: 0

[21, 105, 225, 179, 112, 209]

Step 3: 000101010110100111100001101100110111000011010001

000 101 010 110 100 111 100 001 101 100 110 111 000 011 010 001

Step 4: Frequency of 0: [3, 1, 2, 1, 2, 0, 2, 2, 1, 2, 1, 0, 3, 1, 2, 2]

Step 5 to 8 Map1 filling

[[3, 1, 2, 1], [2, 0, 2, 2], [1, 2, 1, 0], [3, 1, 2, 2]]

Step 9 Map2 filling

[[3, 1, 2, 1], [1, 2, 1, 0], [2, 3, 2, 2], [0, 1, 2, 2]]

Step 10 11 : [3, 1, 2, 0]

data[21, 105, 225, 179]

Step 12 13 14: [1, 2, 1]

Step 15 16 17: 105, 105, 225,

Step 18 19: 0,2

Step 20: 2 and 2 passed to Map1: 105

Step 21: 1

Step 22: binary of 6th right index: 001100

Step 23: Binary with LSB & MSB: 00110001

Step 24: decimal number = 49 **(δ)**

Put the values of σ, β, γ and δ for z = 0

$f(z_i) \mapsto (\sigma_i z_i + \beta_i)/(\gamma_i z_i + \delta_1)$

165*0 + 98 / 232*0 + 49

98/49

2

First value of the S-box is **2**
