# Supplementary material for: Multilevel information fusion for cryptographic substitution box construction based on inevitable random noise in medical imaging
Source: Sci Rep. 2021 Jul 12;11:14282. doi: 10.1038/s41598-021-93344-z (PMC8275796; doi:10.1038/s41598-021-93344-z)
Supplement: Supplementary file 3 — Supplementary Information 3. [file 41598_2021_93344_MOESM3_ESM.pdf]

Multilevel Information Fusion for Cryptographic Substitution Box Construction based on Inevitable Random Noise in Medical Imaging

Muhammad Fahad Khan [1, 2], Khalid Saleem [1], Mohammed Ali Alshara [3], Shariq Bashir [4]

[1] Department of Computer Sciences, Quaid-i-Azam University, Islamabad,
[2] Department of Software Engineering, Foundation University Islamabad, Pakistan
[3] Department of Information Technology, College of Computer and Information Sciences, Imam Mohammad Ibn Saud Islamic University, Riyadh, Saudi Arabia
[4] The College of Arts and Sciences, DMPS Computer Science Section, University of Nizwa, Sultanate of Oman
Correspondence to: **mfkhan@cs.qau.edu.pk**,**fahad.khan@fui.edu.pk**

```
private static Object[] Permutation1 (String[] Th, String[] Sp, String[] Sh, String[] Ip)
  {
     String [] x = Th;                                                          O(1)
     String [] z = Sp;                                                          O(1)
     String [] y = Sh;                                                          O(1)
     String [] w = Ip;                                                          O(1)
     List<String> X = new ArrayList<String>();                                  O(1)
     List<String> Z = new ArrayList<String>();                                  O(1)
     List<String> Y = new ArrayList<String>();                                  O(1)
     List<String> W = new ArrayList<String>();                                  O(1)
     for (int i=0; i<Th.length; i++)                                            O(n)
     {
        if (x[i].charAt(7)== '0')                                               O(1)
        {
           String s1 = x[i].substring(2,8);                                     O(6)
           String s2 = y[i].substring(2,8);                                     O(6)
           String s3 = z[i].substring(2,8);                                     O(6)
           String s4 = w[i].substring(2,8);                                     O(6)
           long count1 = s1.chars().filter(ch -> ch == '0').count();            O(6)
           long count2 = s2.chars().filter(ch -> ch == '0').count();            O(6)
           long count3 = s3.chars().filter(ch -> ch == '0').count();            O(6)
           long count4 = s4.chars().filter(ch -> ch == '0').count();            O(6)
            char tempx = x[i].charAt(7);                                        O(1)
           char tempz = z[i].charAt(7);                                         O(1)
           char tempy = y[i].charAt(7);                                         O(1)
           char tempw = w[i].charAt(7);                                         O(1)
           StringBuilder stringX = new StringBuilder(x[i]);                     O(1)
           StringBuilder stringZ = new StringBuilder(z[i]);                     O(1)
           StringBuilder stringY = new StringBuilder(y[i]);                     O(1)
           StringBuilder stringW = new StringBuilder(w[i]);                     O(1)
           if (count1 > count2)
           {
              stringZ.setCharAt(7, tempx);                                      O(1)
              z[i] = stringZ.toString();                                        O(1)
              stringY.setCharAt(7, tempz)                                       O(1)
              y[i] = stringY.toString();                                        O(1)
```

```
    stringW.setCharAt(7, tempy);                          O(1)
    w[i] = stringW.toString();                            O(1)
    stringX.setCharAt(7, tempw);                          O(1)
    x[i] = stringX.toString();                            O(1)
    tempx = x[i].charAt(7);                               O(1)
    tempz = z[i].charAt(7);                               O(1)
    tempy = y[i].charAt(7);                               O(1)
    tempw = w[i].charAt(7);                               O(1)
    stringZ.setCharAt(7, tempx);                          O(1)
    z[i] = stringZ.toString();                            O(1)
    stringY.setCharAt(7, tempz);                          O(1)
    y[i] = stringY.toString();                            O(1)
    stringW.setCharAt(7, tempy);                          O(1)
    w[i] = stringW.toString();                            O(1)
    stringX.setCharAt(7, tempw);                          O(1)
    x[i] = stringX.toString();                            O(1)
    X.add(x[i]);                                          O(1)
    Y.add(y[i]);                                          O(1)
    Z.add(z[i]);                                          O(1)
    W.add(w[i]);                                          O(1)
}
 else if (count1 < count2)                                O(25)
{
    stringW.setCharAt(7, tempy);
    w[i] = stringW.toString();
    stringX.setCharAt(7, tempw);
    x[i] = stringX.toString();
    stringZ.setCharAt(7, tempx);
    z[i] = stringZ.toString();
    stringY.setCharAt(7, tempz);
    y[i] = stringY.toString();
    tempx = x[i].charAt(7);
    tempz = z[i].charAt(7);
    tempy = y[i].charAt(7);
    tempw = w[i].charAt(7);
    stringW.setCharAt(7, tempy);
    w[i] = stringW.toString();
    stringX.setCharAt(7, tempw);
    x[i] = stringX.toString();
    stringZ.setCharAt(7, tempx);
    z[i] = stringZ.toString();
    stringY.setCharAt(7, tempz);
    y[i] = stringY.toString();
    X.add(x[i]);
    Y.add(y[i]);
    Z.add(z[i]);
    W.add(w[i]);
}
else
{
    if (count3 > count4)                                  O(25)
    {
        stringY.setCharAt(7, tempz);
```

```
            y[i] = stringY.toString();
            stringW.setCharAt(7, tempy);
            w[i] = stringW.toString();
            stringX.setCharAt(7, tempw);
            x[i] = stringX.toString();
            stringZ.setCharAt(7, tempx);
            z[i] = stringZ.toString();
            tempx = x[i].charAt(7);
            tempz = z[i].charAt(7);
            tempy = y[i].charAt(7);
            tempw = w[i].charAt(7);
            stringY.setCharAt(7, tempz);
            y[i] = stringY.toString();
            stringW.setCharAt(7, tempy);
            w[i] = stringW.toString();
            stringX.setCharAt(7, tempw);
            x[i] = stringX.toString();
            stringZ.setCharAt(7, tempx);
            z[i] = stringZ.toString();
            X.add(x[i]);
            Y.add(y[i]);
            Z.add(z[i]);
            W.add(w[i]);
        }
         else if (count3 < count4)                                   O(25)
        {
            stringY.setCharAt(7, tempw);
            y[i] = stringY.toString();
            stringZ.setCharAt(7, tempy);
            z[i] = stringZ.toString();
            stringX.setCharAt(7, tempz);
            x[i] = stringX.toString();
            stringW.setCharAt(7, tempx);
            w[i] = stringW.toString();
            tempx = x[i].charAt(7);
            tempz = z[i].charAt(7);
            tempy = y[i].charAt(7);
            tempw = w[i].charAt(7);
            stringY.setCharAt(7, tempw);
            y[i] = stringY.toString();
            stringZ.setCharAt(7, tempy);
            z[i] = stringZ.toString();
            stringX.setCharAt(7, tempz);
            x[i] = stringX.toString();
            stringW.setCharAt(7, tempx);
            X.add(x[i]);
            Y.add(y[i]);
            Z.add(z[i]);
            W.add(w[i]);
        }
    }
}
 else
{
   String s1 = x[i].substring(2,8);                                    O(6)
```

```
String s2 = y[i].substring(2,8);                                O(6)
String s3 = z[i].substring(2,8);                                O(6)
String s4 = w[i].substring(2,8);                                O(6)
long count1 = s1.chars().filter(ch -> ch == '1').count();       O(6)
long count2 = s2.chars().filter(ch -> ch == '1').count();       O(6)
long count3 = s3.chars().filter(ch -> ch == '1').count();       O(6)
long count4 = s4.chars().filter(ch -> ch == '1').count();       O(6)
char tempx = x[i].charAt(7);                                    O(1)
char tempz = z[i].charAt(7);                                    O(1)
char tempy = y[i].charAt(7);                                    O(1)
char tempw = w[i].charAt(7);                                    O(1)
StringBuilder stringX = new StringBuilder(x[i]);                O(1)
StringBuilder stringZ = new StringBuilder(z[i]);                O(1)
StringBuilder stringY = new StringBuilder(y[i]);                O(1)
StringBuilder stringW = new StringBuilder(w[i]);                O(1)
 if (count1 > count2)                                           O(25)
{
   stringZ.setCharAt(7, tempx);
   z[i] = stringZ.toString();
   stringY.setCharAt(7, tempz);
   y[i] = stringY.toString();
   stringW.setCharAt(7, tempy);
   w[i] = stringW.toString();
   stringX.setCharAt(7, tempw);
   x[i] = stringX.toString();
   tempx = x[i].charAt(7);
   tempz = z[i].charAt(7);
   tempy = y[i].charAt(7);
   tempw = w[i].charAt(7);
   stringZ.setCharAt(7, tempx);
   z[i] = stringZ.toString();
   stringY.setCharAt(7, tempz);
   y[i] = stringY.toString();
   stringW.setCharAt(7, tempy);
   w[i] = stringW.toString();
   stringX.setCharAt(7, tempw);
   x[i] = stringX.toString();
   X.add(x[i]);
   Y.add(y[i]);
   Z.add(z[i]);
   W.add(w[i]);
}
    else if (count1 < count2)                                   O(25)
{
   stringW.setCharAt(7, tempy);
   w[i] = stringW.toString();
   stringX.setCharAt(7, tempw);
   x[i] = stringX.toString();
   stringZ.setCharAt(7, tempx);
   z[i] = stringZ.toString();
   stringY.setCharAt(7, tempz);
   y[i] = stringY.toString();
   tempx = x[i].charAt(7);
   tempz = z[i].charAt(7);
```

```
    tempy = y[i].charAt(7);
    tempw = w[i].charAt(7);
    stringW.setCharAt(7, tempy);
    w[i] = stringW.toString();
    stringX.setCharAt(7, tempw);
    x[i] = stringX.toString();
    stringZ.setCharAt(7, tempx);
    z[i] = stringZ.toString();
    stringY.setCharAt(7, tempz);
    y[i] = stringY.toString();
    X.add(x[i]);
    Y.add(y[i]);
    Z.add(z[i]);
    W.add(w[i]);
}
else
{
    if (count3 > count4)                                          O(25)
    {
        stringY.setCharAt(7, tempz);
        y[i] = stringY.toString();
        stringW.setCharAt(7, tempy);
        w[i] = stringW.toString();
        stringX.setCharAt(7, tempw);
        x[i] = stringX.toString();
        stringZ.setCharAt(7, tempx);
        z[i] = stringZ.toString();
        tempx = x[i].charAt(7);
        tempz = z[i].charAt(7);
        tempy = y[i].charAt(7);
        tempw = w[i].charAt(7);
        stringY.setCharAt(7, tempz);
        y[i] = stringY.toString();
        stringW.setCharAt(7, tempy);
        w[i] = stringW.toString();
        stringX.setCharAt(7, tempw);
        x[i] = stringX.toString();
        stringZ.setCharAt(7, tempx);
        z[i] = stringZ.toString();
        X.add(x[i]);
        Y.add(y[i]);
        Z.add(z[i]);
        W.add(w[i]);
    }
     else if (count3 < count4)                                    O(25)
    {
        stringY.setCharAt(7, tempw);
        y[i] = stringY.toString();
        stringZ.setCharAt(7, tempy);
        z[i] = stringZ.toString();
        stringX.setCharAt(7, tempz);
        x[i] = stringX.toString();
        stringW.setCharAt(7, tempx);
        w[i] = stringW.toString();
        tempx = x[i].charAt(7);
```

```
                tempz = z[i].charAt(7);
                tempy = y[i].charAt(7);
                tempw = w[i].charAt(7);
                stringY.setCharAt(7, tempw);
                y[i] = stringY.toString();
                stringZ.setCharAt(7, tempy);
                z[i] = stringZ.toString();
                stringX.setCharAt(7, tempz);
                x[i] = stringX.toString();
                stringW.setCharAt(7, tempx);
                X.add(x[i]);
                Y.add(y[i]);
                Z.add(z[i]);
                W.add(w[i]);
              }
            }
          }
        }
        return new Object[] {X,Z,Y,W};                                   O(1)
      }
```

$F(n) = 135n + 9 \quad \ldots \qquad (1)$

$O(1) = n$

```
private static int[] Layer3 (int [] Data, List<String> DataB)
  {
    int Length = Data.length/6;                                          O(1)
    int [] ABCD = new int[Length];                                       O(1)
    int x=0;                                                             O(1)
    for (int i=0; i<Length; i++)                                         O(1)
    {
      int k = 0;                                                         O(1)
      int j = x;                                                         O(1)
      List <String> dataBin = new ArrayList<>();                         O(1)
      int [] dataInt = new int[6];                                       O(1)

      while(k<6)                                                         O(6)
      {
        dataInt[k] = Data[j];                                            O(1)
        dataBin.add(DataB.get(j));                                       O(1)
        k++;                                                             O(1)
        j++;                                                             O(1)
      }
      x=x+6;                                                             O(1)
      Object [] mp = Generation(dataBin);                                O(1)
      int [][] map1 = (int[][]) mp[0];                                   O(1)
      int [][] map2 = (int[][]) mp[1];                                   O(1)
      int[] ArraysIndex = Index_Generation(map2);                        O(1)
      String s = "";                                                     O(1)
      for (int f=0; f<6; f++)                                            O(6)
      {
        if (!checkNumberExists(s, dataInt[f]))                           O(1)
```

```
        {
           s = s + dataInt[f] + "#";                                    O(1)
        }
     }
     String [] ary = s.split("#");                                      O(1)
     if (ary.length<4)                                                  O(1)
           continue;                                                    O(1)
     int [] data = new int[4];                                          O(1)
     for(int f=0; f<4; f++)                                             O(4)
     {
        data[f] = Integer.parseInt(ary[f]);                             O(1)
     }
     int number = Levels(ArraysIndex,data,map1,map2);                   O(1)
     ABCD[i] = number;                                                  O(1)
  }
  return ABCD;                                                          O(1)
}
```

F(n) = 3 + n (4+6(4) + 5 + 6 (2) + 4 + 4(1) + 2) .. (2)
F(n) = 3 + 55n
O(1) = n

```
public static Object[] Permutation2(String[] Th, String[] Sp, String[] Sh, String[] Ip)
{
   String[] x = Th;                                                     O(1)
   String[] z = Sp;                                                     O(1)
   String[] y = Sh;                                                     O(1)
   String[] w = Ip;                                                     O(1)
    for (int i=1; i<Th.length-1; i++)                                   O(n)
   {
      char Bit1 = z[i].charAt(1);                                       O(1)
      char Bit2 = z[i+1].charAt(0);                                     O(1)
      char Bit3 = z[i-1].charAt(2);                                     O(1)
      char Bit4 = w[i+1].charAt(3);                                     O(1)
      char Bit5 = w[i-1].charAt(4);                                     O(1)
      char Bit6 = y[i+1].charAt(5);                                     O(1)
      char Bit7 = y[i-1].charAt(6);                                     O(1)
      char Bit8 = y[i].charAt(7);                                       O(1)
      String K = new String(new char[]{Bit1,Bit2,Bit3,Bit4,Bit5,Bit6,Bit7,Bit8});O(1)
      int num = Integer.parseInt(K.substring(0,6),2);                   O(7)
      char temp='0';                                                    O(1)
      StringBuilder st;                                                 O(1)
      if(K.charAt(7)== '0')                                             O(1)
      {
         for (int j=0; j<num; j++)                                      O(255)
         {
            String [] tempX = new String[x.length];
            for (int k=0; k<x.length; k++)                              O(n)
            {
               st = new StringBuilder(x[k]);                            O(1)
               if (k==0)                                                O(4)
               {
                  temp = x[x.length-1].charAt(7);
                  st.setCharAt(7, temp);
                  tempX[k] = st.toString();
```

```
            }
            else
            {
               temp = x[k-1].charAt(7);
               st.setCharAt(7, temp);
               tempX[k] = st.toString();
            }
         }
         x = tempX;
      }
   }
   else
   {
      for (int j=0; j<num; j++)                                    O(255)
      {
         String [] tempY = new String[y.length];
         for (int k=0; k<y.length; k++)                             O(n)
         {
            st = new StringBuilder(y[k]);                           O(1)
            if (k==0)                                               O(4)
            {
               temp = y[y.length-1].charAt(7);
               st.setCharAt(7, temp);
               tempY[k] = st.toString();
            }
            else
            {
               temp = y[k-1].charAt(7);
               st.setCharAt(7, temp);
               tempY[k] = st.toString();
            }
         }
         y = tempY;
      }
   }
   if(K.charAt(6)== '0')
   {
     for (int j=0; j<num; j++)                                        O(255)
      {
         String [] tempZ = new String[z.length];
         for (int k=0; k<z.length; k++)                                 O(n)
         {
            st = new StringBuilder(z[k]);
            if (k==0)                                                   O(4)
            {
               temp = z[z.length-1].charAt(7);
               st.setCharAt(7, temp);
               tempZ[k] = st.toString();
            }
            else
            {
               temp = z[k-1].charAt(7);
               st.setCharAt(7, temp);
               tempZ[k] = st.toString();
            }
```

```
                }
                z = tempZ;
            }
        }
        else
        {
            for (int j=0; j<num; j++)                                   O(255)
            {
                String [] tempW = new String[w.length];
                for (int k=0; k<w.length; k++)                          O(n)
                {
                    st = new StringBuilder(w[k]);
                    if (k==0)                                           O(4)
                    {
                        temp = w[w.length-1].charAt(7);
                        st.setCharAt(7, temp);
                        tempW[k] = st.toString();
                    }
                    else
                    {
                        temp = w[k-1].charAt(7);
                        st.setCharAt(7, temp);
                        tempW[k] = st.toString();
                    }
                }
                w = tempW;
            }
        }
    }
        return new Object[] {x,z,y,w};                                  O(1)
}
```

$F(n) = 4 + 18n\ (\ 255(n+4)) + 1$ ... (3)

$F(n) = 5 + 18n\ (\ 255n+1020)$

$F(n) = 5 + 4095n^2 + 18360n$

$F(n) = 4095n^2 + 18360n+5$

$O(n) = n^2$
